# Risk factors and biomarkers of non-alcoholic fatty liver disease: an observational cross-sectional population survey

**DOI:** 10.1136/bmjopen-2017-019974

**Published:** 2018-04-05

**Authors:** Xiao-Yu Hu, Yun Li, Long-Quan Li, Yuan Zheng, Jia-Hong Lv, Shu-Chun Huang, Weinong Zhang, Liang Liu, Ling Zhao, Zhuiguo Liu, Xiu-Ju Zhao

**Affiliations:** 1 School of Biology and Pharmaceutical Engineering, Wuhan Polytechnic University, Wuhan, China; 2 Hubei Key Laboratory of Lipid Chemistry and Nutrition of Oil, Oil Crops Research Institute of the Chinese Academy of Agricultural Sciences, Wuhan, China; 3 Department of Nutrition and Food Science, Texas A&M University, College Station, Texas, USA

**Keywords:** epidemiology, hepatology, public health

## Abstract

**Objective:**

Non-alcoholic fatty liver disease (NAFLD) is a major public health burden in China, and its prevalence is increasing. This study aimed to determine the risk factors and biomarkers of NAFLD.

**Design:**

An observational cross-sectional primary survey.

**Setting:**

Central China.

**Participants:**

The study included 1479 participants aged over 18 and below 80 years, not currently being treated for cancer or infectious disease or no surgery in the previous year, and no history of cancer or an infectious disease. Participants underwent clinical examination, metabolomic assay and anthropometric assessment. Univariate and logistic regression analyses were used to evaluate associations between covariates and NAFLD.

**Main outcome measures:**

Risk factors and metabolic biomarkers including sex, body mass index, hypertension, body fat ratio, blood triglycerides, blood fasting glucose, liver enzyme elevation, uric acid and oleic acid-hydroxy oleic acid (OAHOA).

**Results:**

Data from the 447 participants (mean age 44.3±11.9 years) were analysed, and the prevalence of NAFLD was 24.7%. Male sex (OR 3.484, 95% CI 2.028 to 5.988), body mass index ≥24 kg/m^2^ (OR 8.494, 95% CI 5.581 to 12.928), body fat ratio (≥25 for women, ≥20 for men) (OR 1.833, 95% CI 1.286 to 2.756), triglycerides ≥1.7 mmol/L (OR 1.340, 95% CI 1.006 to 1.785), fasting glucose ≥6.1 mmol/L (OR 3.324, 95% CI 1.888 to 5.850), blood pressure ≥140/90 mm Hg or antihypertensive drug treatment (OR 1.451, 95% CI 1.069 to 1.970), uric acid (≥357 μmol/L for women, ≥416 μmol/L for men) (OR 2.755, 95% CI 2.009 to 3.778) and OAHOA (<5 nmol/L) (OR 1.340, 95% CI 1.006 to 1.785) were independent predictors of NAFLD (all P<0.05). These results were verified by all 1479 participants.

**Conclusions:**

NAFLD was common among the study participants. In particular, NAFLD was correlated with uric acid. We identified OAHOA as a novel marker of NAFLD prevalence. It provides a reference on the prevention of NAFLD and related metabolic diseases with the rapid urbanisation, technological advancement and population ageing in China over the recent decades.

**Strengths and limitations of this study**

- Risk factors of non-alcoholic fatty liver disease (NAFLD) in central China.
- Biomarkers of NAFLD using metabolomics.
- Prediction ability of metabolic markers using receiver operating characteristic curve.
- No information on education.

## Introduction

China has the world’s largest population and is undergoing rapid economic growth and social reform. This advancement has paralleled demographic, lifestyle and cultural changes, which have exerted notable effects on the health profile of China’s residents and placed significant constraints on the country’s healthcare system.^1^

Such changes are apparent in major cities of central China, such as Wuhan. The prevalence of non-alcoholic fatty liver disease (NAFLD) is increasing, now the most common chronic disease among Chinese adults in China, and the rates exceed those of equally important epidemics, including obesity, hypertension and type 2 diabetes. NAFLD is followed by an increase in overall cardiovascular morbidity and mortality.^2–4^ It has been estimated that NAFLD will be the most frequent indicator for liver transplantation and regeneration in the coming decades.^5–7^ Additionally, because population ageing and the continual increase in obesity and hypertension rates over the recent decades, the prevalence and impact of NAFLD in China are expected to increase. Accordingly, NAFLD is a major public health burden.

The reported prevalence of NAFLD among Chinese adults ranges from 15% to 30%.^5–7^ NAFLD prevalence increases with age, most notably from the fourth decade of life onwards (40–60 years of age).^5 6 8 9^ Because of endocrine function variations and fat redistribution, the prevalence and risk factors for NAFLD may vary across different ages and between male and female populations.^10–13^ However, the association of age and sex with NAFLD in central China is still unclear. Furthermore, the association of hypertension, obesity, hyperlipidaemia, insulin resistance and diabetes with NAFLD has been widely evaluated in adult cohorts,^14–16^ and these metabolic traits are well-accepted risk factors at present for NAFLD. Nonetheless, limited data are available on the association of NAFLD with other covariant traits.^17 18^ Metabolomics provides integrated information on biological status and can investigate molecular variations with disease phenotyping^19 20^; several markers for NAFLD have been identified previously.^5 6^ Sphingolipids (SLs) and branched fatty acid esters of hydroxy fatty acids (FAHFAs) have been reported to protect against autoimmune and allergic disorders and type 2 diabetes, respectively^21 22^; the effects of SLs and FAHFAs on NAFLD are largely unclear.

Our present study was aimed at investigating the prevalence of NAFLD, elucidating the association of NAFLD with age, sex, metabolic factors and markers of liver injury, and identifying novel metabolites associated with NAFLD risk, via clinical examination, anthropometric assessment and targeted metabolomics.

## Methods

### Study population

All participants provided written informed consent prior to enrolment. The Wuhan study is an observational cross-sectional study of participants recruited from the Wuhan municipality in central China. Participants were recruited from routine health check-ups to assess the utility of health check-ups in determining the prevalence of and risk factors for NAFLD. Each participant completed a comprehensive interview and clinical examination that involved the collection of fasting blood and urine samples, abdominal ultrasonography, and anthropometric assessment.

### Interview

The interview, followed by the clinical examination, was performed by a physician and was designed to obtain information on demographic characteristics, medical history and comorbid conditions. Participants aged over 18 and below 80 years were included if they were not currently being treated for cancer or infectious disease or had undergone surgery in the previous year, and if they had no history of cancer or an infectious disease.

### Biochemistry

Fasting blood and urine samples were collected on clinical examination. Blood alanine aminotransferase (ALT), alkaline phosphatase, aspartate aminotransferase (AST), gamma-glutamyl transferase, glucose, total bilirubin, triglycerides and uric acid levels were measured using automatic enzymatic procedures (Nanjing Jiancheng Sci-tech, China). Hepatitis B surface antigen (HBsAg), triiodothyronine, thyroxine (T4) and thyroid-stimulating hormone were examined via automatic immunoassays (Roche Diagnostics, Mannheim, Germany).

### Diagnosis of NAFLD

Abdominal ultrasonography was performed on all participants by accredited medical technicians using a Hitachi HI VISION 900 ultrasound machine. Images were evaluated by a senior pathologist. NAFLD was diagnosed by the technician according to ultrasonography.^23–25^ Participants with any of the following possible secondary causes of fatty liver were excluded from the current analyses: (1) excessive alcohol intake,^26^ (2) anti-hepatitis C virus (HCV) or positive HBsAg, or (3) use of drugs historically for treating fatty liver (ie, amiodarone, corticosteroids, methotrexate or tamoxifen).^13^

### Metabolic covariates

Anthropometric measurements were performed by accredited nurses. Body mass index (BMI) was defined as the measured weight (kg) divided by height squared (m^2^). Body fat ratio (BFR) was equal to fat weight (kg) divided by body weight (kg). The average of two resting blood pressure (BP) measurements at a single visit after a 10 min break was used for analysis.

Metabolic traits were defined as the following: obesity (BMI ≥28 kg/m^2^), hypertension (BP ≥140/90 mm Hg or antihypertensive drug treatment), BFR ≥25 for women or ≥20 for men, blood triglycerides ≥1.7 mmol/L, blood fasting glucose ≥6.1 mmol/L, liver enzyme elevation (AST ≥40 U/L or ALT ≥40 U/L), uric acid ≥357 μmol/L for women or ≥416 μmol/L for men, or impaired oleic acid-hydroxy oleic acid (OAHOA) (<5 nmol/L).

### Targeted Liquid Chromatography/Mass Spectrometry analysis of SLs and FAHFAs

To identify SLs,^21^ silica column-affectionate lipid fractions were analysed by reverse-phase high-performance liquid chromatography-tandem mass spectrometry (HPLC-MS)/MS (Agilent C18 column connected with Thermo Scientific LTQXL; gradients of water to methanol, 10:90 to 0:100) to identify spots present in both control and liver disease. Identified lipid species of interest were purified by reverse-phase HPLC (Varian Prostar/Agilent C18 column) and subjected to structural analysis.

FAHFAs were measured by HPLC-MS^22^ (an Agilent 6410 Triple Quad liquid chromatography (LC)/mass spectrometry (MS) via multiple reaction monitoring (MRM) in a negative ionisation mode). Briefly, extracted and fractionated samples were dissolved in 25 mL MeOH; 10 mL was injected for further analysis. A Luna C18(2) (Phenomenex) column was used with an in-line filter (Phenomenex). Distinct FAHFAs were dissolved via isocratic flow (0.2 mL/min for 120 min, solvent: 93:7 MeOH:H_2_O, 5 mM ammonium acetate, 0.01% ammonium hydroxide). Transitions for OAHOAs were m/z 561.5 → m/z 281.2 (collision energy (CE)=30 V), and m/z 561.5 → m/z 279.2 (CE=25 V).

### Statistical analyses

Baseline characteristics were analysed using descriptive statistics. U tests, Χ^2^ tests or Wilcoxon rank-sum tests (for medians) and Student’s t-tests (for means) were used to evaluate the significance of differences in the distribution of categorical data and continuous data, respectively. To examine the relations between traits and NAFLD, we performed binary or multiple logistic regression analyses. We calculated the area under the receiver operating curve to assess prediction ability of metabolic markers. A P value of <0.05 was considered statistically significant unless stated otherwise. Data analyses were performed using SPSS V.17.0.

## Results

### Study population

In total, 1500 participants were enrolled. Twenty-one participants were excluded due to absence of clinical data. Thus, a total of 1479 study participants were included in the final analysis, of whom 447 reported their age and had no more than one disease, if any (denoted as the ‘subset’). Patients’ general characteristics are shown in table 1. Men accounted for 40% of the study population (38% of the subset). Participants’ mean age was 44.3±11.9 (range 20–74) years.

**Table 1:** Participant characteristics

Indicators	Total	NAFLD	No NAFLD	P value
(A) Subset				
n	447	102 (22.8%)	345 (77.2%)	
Age (years)	44.3±11.9	44.6±10.8	44.0±12.1	0.609
Age range	4.0±1.2	4.0±1.2	3.9±1.2	0.608
Obesity	235 (51.8%)	94	141	<0.001
Body fat	309 (68.1%)	89	220	<0.001
Blood lipids	87 (19.2%)	21	66	0.745
Hypertension	55 (12.1%)	23	32	0.003
Male	171 (38.3%)	64	107	<0.001
Elevated liver enzymes	18 (4.0%)	7	11	0.174
(B) Total study population				
n	1479	365 (24.7%)	1114 (75.3%)	
Obesity	809 (54.7%)	334	475	<0.001
Body fat	1003 (67.8%)	309	694	<0.001
Blood lipid	458 (30.9%)	163	295	<0.001
High blood glucose	71 (4.8%)	43	28	<0.001
Hypertension	314 (21.2%)	129	185	<0.001
Abnormal uric acid	83 (5.6%)	38	45	<0.001
Male	590 (39.8%)	214	376	<0.001
Impaired liver function	32 (0.2%)	12	20	0.142
Elevated liver enzymes	110 (0.7%)	53	57	<0.001

Age ranges of 20–30, 30–40, 40–50, 50–60 and ≥60 are 2, 3, 4, 5 and 6.

Conditions were defined as follows: obesity (body mass index ≥24 kg/m^2^), hypertension (blood pressure ≥140/90 mm Hg or antihypertensive drug treatment), body fat ratio ≥25 for women or ≥20 for men, blood triglycerides ≥1.7 mmol/L, blood fasting glucose ≥5.6 mmol/L, impaired liver function (positive HBsAg), liver enzyme elevation (AST ≥40 U/L or ALT ≥40 U/L), and uric acid ≥357 µmol/L for women or ≥416 µmol/L for men.

Mean values are provided with SD, unless otherwise noted as n (%). Differences between participants with and without NAFLD were evaluated with t-tests or the Wilcoxon-Mann-Whitney test for continuous variables and the χ^2^ test for categorical variables.

ALT, alanine aminotransferase; AST, aspartate aminotransferase; HBsAg, hepatitis B surface antigen; NAFLD, non-alcoholic fatty liver disease.

### Prevalence of NAFLD

The prevalence of NAFLD, as determined by ultrasound and biopsy, was 24.7% (22.8% in the subset).

The peak prevalence of NAFLD was 26.4% and 26.3% in this study between the ages of 30–40 years and 50–60 years, respectively (table 2).

**Table 2:** NAFLD prevalence across age range

Age range (years)	Participants, n	Participants with NAFLD, n	NAFLD prevalence,%	U test
20–30	64	10	15.6	1.22
30–40	102	27	26.4	0.75
40–50	120	26	21.7	0.18
50–60	111	30	27.0	1.00
≥60	50	9	18.0	0.64
Total	447	102	22.8	1.64 (α=0.1)

NAFLD, non-alcoholic fatty liver disease.

The prevalence of NAFLD demonstrated an increasing trend with advancing age (OR 1.049, P=0.607), and after adjustment for sex and metabolic features NAFLD tended to be inversely correlated with age (OR 0.844, 95% CI 0.667 to 1.068; P=0.157). NAFLD was diagnosed in 37.4% of men and 13.8% of women (P=0.084). In multivariate analysis, after adjustment for metabolic features and age, male sex was associated with NAFLD (OR 3.484, 95% CI 2.028 to 5.988; P<0.001; table 3A).

**Table 3:** Multivariate adjusted models for liver disease subtypes

Variables	OR	P value
(A) Subset		
Sex (male)	3.484	<0.001
Obesity	11.738	<0.001
Body fat	2.285	0.033
Elevated liver enzyme	2.237	0.105
Hypertension	1.865	0.089
(B) Whole population		
Sex (male)	2.646	<0.001
Obesity	8.494	<0.001
Body fat	1.833	0.001
Blood lipid	1.340	0.046
Blood glucose	3.324	<0.001
Impaired liver function	1.859	0.094
Elevated liver enzyme	3.150	<0.001
Hypertension	1.451	0.017

Conditions were defined as follows: female sex=1, obesity (body mass index ≥28 kg/m^2^), hypertension (blood pressure ≥140/90 mm Hg or antihypertensive drug treatment), body fat ratio ≥25 for women or ≥20 for men, blood fasting glucose ≥6.1 mmol/L, and liver enzyme elevation (AST ≥40 U/L or ALT ≥40 U/L).

ORs and the corresponding P values derived from binary or multiple logistic regression analyses using SPSS V.17.0 software.

ALT, alanine aminotransferase; AST, aspartate aminotransferase.

### Association between NAFLD and metabolic features

Among metabolic covariates, obesity and hypertension occurred more frequently in participants with advancing age (OR 1.471 and 1.822, respectively; P<0.001). Increased body fat was more prevalent in women than in men (OR 2.042, P<0.001 (OR 1.754, P=0.007 in the subset)), and obesity (OR 0.537, P<0.001), increased blood lipids (OR 0.829, P<0.101) and hypertension (OR 0.769, P<0.041) were more prevalent in men than in women (OR 0.472, P<0.001; OR 0.902, P<0.673; and OR 0.602, P<0.080, respectively, in the subset).

In univariate analysis, all metabolic and anthropometric traits were significantly associated with NAFLD. In logistic regression analysis, after adjustment for sex, BMI ≥24 kg/m^2^ (OR 8.494, 95% CI 5.581 to 12.928; P<0.001), BFR ≥25 for women and ≥20 for men (OR 1.833, 95% CI 1.286 to 2.756; P=0.001), triglycerides ≥1.7 mmol/L (OR 1.340, 95% CI 1.006 to 1.785; P=0.046), fasting glucose ≥6.1 mmol/L (OR 3.324, 95% CI 1.888 to 5.850; P<0.001), BP ≥140/90 mm Hg or antihypertensive drug treatment (OR 1.451, 95% CI 1.069 to 1.970; P=0.017), uric acid ≥357 μmol/L for women and ≥416 μmol/L for men (OR 2.755, 95% CI 2.009 to 3.778; P<0.001), and total OAHOA <5 nmol/L (OR 1.340, 95% CI 1.006 to 1.785; P=0.046) (after adjustment for age ranges and sex in the subset, ORs for BMI, BFR and BP were 11.738 (95% CI 5.193 to 26.530, P<0.001), 2.285 (95% CI 1.067 to 4.893, P=0.033) and 1.865 (95% CI 0.910 to 3.824, P=0.089), respectively) were independent predictors of NAFLD (table 3A,B and table 4). The prevalence of NAFLD demonstrated an increasing trend with lowering sphingosines (OR 1.448, P=0.156; table 4). Substitution of age ranges for continuous data (ie, absolute age) in logistic regression did not modify the relationships. When each metabolic trait was analysed independently, after adjustment for age and sex, ORs increased for obesity and hypertension with increasing age, and ORs increased for BFR with increasing age for patients aged <50 years and decreased for patients aged ≥50 years. However, significant interactions were only observed between age and obesity (P<0.001), and between age and hypertension (P<0.001).

**Table 4:** Metabolites associated with non-alcoholic fatty liver disease

Metabolite	OR	P value	AUC	P value
Uric acid	2.755	<0.001	0.579	<0.001
Sphingosine	1.448	0.156	0.489	0.760
OAHOA	1.340	0.046	0.612	0.001

Conditions were defined as sphingosine <2 nmol/L and OAHOA (oleic acid-hydroxy oleic acid) <5 nmol/L.

ORs, area under curve (AUC) and the corresponding P values derived from binary or multiple logistic regression, or receiver operating characteristic curve analyses using SPSS V.17.0 software.

### Association between NAFLD and liver enzymes

Participants with NAFLD had higher liver enzyme levels than participants without this condition (P<0.001). Abnormal liver enzymes were also significantly associated with NAFLD, independent of sex (OR 3.150, P<0.001). Normal liver enzyme levels, defined according to local guidelines (ALT <40 U/L), were found in 85% of participants with NAFLD.

## Discussion

Our results demonstrated that NAFLD was strongly associated with metabolic traits including higher body fat, obesity, hyperlipidaemia and impaired fasting glucose. We observed a higher prevalence of NAFLD in men than in women. We conformed the association of impaired uric acid and abnormal liver enzymes with NAFLD, and further identified impaired total OAHOA as a novel biomarker of NAFLD prevalence.

The average and peak prevalence of NAFLD in this study were 24.7% and more than 26%, respectively, close to the global prevalence of NAFLD, which was 25.24%, with the highest prevalence in the Middle East (31.79%) and South America (30.45%), and the lowest in Africa (13.48%).^27^ The average and peak prevalence of NAFLD in Shanghai^5^ were 20.82% and 28.44%, respectively, and those in Guangdong^6^ were 17.2% and 27.4%, respectively. The former study was conducted from 2002 to 2003, and the latter in 2005. Our study, performed in 2010, revealed that the average prevalence is increasing, and the peak prevalence occurred at a younger age (30–40 years) than was reported by the previous studies in Shanghai (age of 60–69),^5^ Guangdong (age of 60–69)^6^ and worldwide (mean age of 70–79).^27^ Thus, more attention should be given to the health of people in their 30s, when many marry and adopt lifestyle changes, especially in urban China. Unfortunately, we did not have access to information on participants’ activity levels, and thus the effects of physical activity on NAFLD were not assessed. The reasons underlying the observed earlier peak prevalence require further investigation.

We observed that male sex was a risk factor for NAFLD. This finding differs from the results of the Guangdong study^6^ and the Rotterdam study.^13^ However, this finding is similar to that of the Shanghai study.

There are several possibilities for this discrepancy. First, the observed sex difference in NAFLD prevalence may be the result of body fat, which is a risk factor for NAFLD and was higher in men than in women in this study. Therefore, male sex may promote NAFLD through mediators such as increased body fat and hormonal change. Second, the sex difference in NAFLD may also result from a lower prevalence of boosted serum triglycerides, glucose and hypertension in women compared with men. A lower prevalence of boosted serum triglycerides, glucose and hypertension in women was reported in a previous study.^28^ However, a causal relationship between NAFLD and these serum metabolic markers, as suggested by previous research,^29 30^ could not be established in our research due to its cross-sectional characteristics. Third, abnormal liver enzymes were more prevalent in men than in women, and both were independent predictors of NAFLD.

We observed that the associations of the identified metabolites with NAFLD risk were pronounced in the Wuhan cohort. These metabolites might play a major role in the development of onset NAFLD. Uric acid was particularly associated with NAFLD in men with type 2 diabetes, besides insulin resistance and other metabolic factors.^31^ High concentrations of uric acid induce the accumulation of reactive oxygen species in hepatocyte mitochondria, ultimately leading to mitochondrial damage,^32^ and uric acid was positively correlated with other metabolic traits and was a risk factor for type 2 diabetes mellitus with NAFLD.^33^

Serum uric acid (SUA) is produced by purine nucleotide catabolism.^34^ SUA subjects have an about twofold higher risk of NAFLD, as shown by ultrasonography. The risk may be independent of age, sex and obesity (indicated by BMI and waist circumference). SUA is an independent risk factor in NAFLD in both Uyghurs and Hans in north-western China.^35^ Among participants with elevated SUA levels, women likely showed a greater risk of NAFLD than men.^36^

OAHOAs are present in humans, which are an endogenous branched FAHFAs, and levels are reduced with obesity and insulin resistance. OAHOA levels in serum are correlated highly with whole-body insulin sensitivity. OAHOAs are endogenous GPR120 ligands and may also exert anti-inflammatory effects in vivo through fatty acid-activated G-protein-coupled receptors (GPCRs), such as GPR120. Androgen-induced protein 1 (AIG1) and androgen-dependent TFPI-regulating protein (ADTRP), atypical integral membrane hydrolases, degrade bioactive FAHFAs,^37^ and branched FAHFAs are preferred substrates of MODY8, a protein carboxyl ester lipase.^38^ Changes in the concentrations of these anti-inflammatory metabolites and in their signalling networks may provide new targets for metabolic and inflammatory diseases.^22^ GLUT4 expression and levels of FAHFAs with antidiabetic and anti-inflammatory effects regulate de novo lipogenesis in adipocytes.^39^ Compared with healthy controls, FAHFAs are significantly decreased in the sera of patients with breast cancer.^40^ Branched FAHFAs protect against colitis by controlling gut innate and adaptive immune responses.^41^

Our study also has some potential limitations. First, we cannot rule out the possibility of biased selection, since non-responders may have had different morbidities. Volunteers in research studies tend to be better educated, healthier and have better lifestyles.^42^ Consequently, estimations of the prevalence of NAFLD may have been biased. Second, only 447 of the 1479 participants provided information on their ages, and we did not obtain data on participants’ educational levels or incomes. Third, specific OAHOA isomers^22^ require further study.

## Conclusions

NAFLD is common in the study population. In this observational cross-sectional primary survey, we found that NAFLD was associated with male sex, and the peak prevalence occurred at a younger age. In particular, NAFLD was correlated with uric acid. Moreover, we identified OAHOA as a novel biomarker of NAFLD. Further studies are needed to explore the potential factors contributing to these relationships. However, this study is valuable in that it provides a reference on the prevention of NAFLD and related metabolic diseases with the rapid urbanisation, technological advancement and population ageing in China.

## Supplementary Material

Reviewer comments

Author's manuscript

## References

[1] Yusuf S, Reddy S, Ounpuu S, et al Global burden of cardiovascular diseases: part I: general considerations, the epidemiologic transition, risk factors, and impact of urbanization. Circulation 2001;104:2746–53. 10.1161/hc4601.099487 11723030

[2] Dunn W, Xu R, Wingard DL, et al Suspected nonalcoholic fatty liver disease and mortality risk in a population-based cohort study. Am J Gastroenterol 2008;103:2263–71. 10.1111/j.1572-0241.2008.02034.x 18684196 PMC2574666

[3] Ong JP, Pitts A, Younossi ZM Increased overall mortality and liver-related mortality in non-alcoholic fatty liver disease. J Hepatol 2008;49:608–12. 10.1016/j.jhep.2008.06.018 18682312

[4] Targher G, Day CP, Bonora E Risk of cardiovascular disease in patients with nonalcoholic fatty liver disease. N Engl J Med Overseas Ed 2010;363:1341–50. 10.1056/NEJMra0912063 20879883

[5] Fan JG, Zhu J, Li XJ, et al Prevalence of and risk factors for fatty liver in a general population of Shanghai, China. J Hepatol 2005;43:508–14. 10.1016/j.jhep.2005.02.042 16006003

[6] Zhou YJ, Li YY, Nie YQ, et al Prevalence of fatty liver disease and its risk factors in the population of South China. World J Gastroenterol 2007;13:6419–24. 10.3748/wjg.v13.i47.6419 18081233 PMC4205463

[7] Fan JG, Farrell GC Epidemiology of non-alcoholic fatty liver disease in China. J Hepatol 2009;50:204–10. 10.1016/j.jhep.2008.10.010 19014878

[8] Schwimmer JB, Deutsch R, Kahen T, et al Prevalence of fatty liver in children and adolescents. Pediatrics 2006;118:1388–93. 10.1542/peds.2006-1212 17015527

[9] Ayonrinde OT, Olynyk JK, Beilin LJ, et al Gender-specific differences in adipose distribution and adipocytokines influence adolescent nonalcoholic fatty liver disease. Hepatology 2011;53:800–9. 10.1002/hep.24097 21374659

[10] Roy AK, Chatterjee B Sexual dimorphism in the liver. Annu Rev Physiol 1983;45:37–50. 10.1146/annurev.ph.45.030183.000345 6189449

[11] Sepe A, Tchkonia T, Thomou T, et al Aging and regional differences in fat cell progenitors - a mini-review. Gerontology 2011;57:66–75. 10.1159/000279755 20110661 PMC3031153

[12] Takasugi M, Hayakawa K, Arai D, et al Age- and sex-dependent DNA hypomethylation controlled by growth hormone in mouse liver. Mech Ageing Dev 2013;134:331–7. 10.1016/j.mad.2013.05.003 23707638

[13] Koehler EM, Schouten JN, Hansen BE, et al Prevalence and risk factors of non-alcoholic fatty liver disease in the elderly: results from the Rotterdam study. J Hepatol 2012;57:1305–11. 10.1016/j.jhep.2012.07.028 22871499

[14] Marchesini G, Brizi M, Morselli-Labate AM, et al Association of nonalcoholic fatty liver disease with insulin resistance. Am J Med 1999;107:450–5. 10.1016/S0002-9343(99)00271-5 10569299

[15] Pagano G, Pacini G, Musso G, et al Nonalcoholic steatohepatitis, insulin resistance, and metabolic syndrome: further evidence for an etiologic association. Hepatology 2002;35:367–72. 10.1053/jhep.2002.30690 11826410

[16] Cui J, Chen CH, Lo MT, et al Shared genetic effects between hepatic steatosis and fibrosis: A prospective twin study. Hepatology 2016;64:1547–58. 10.1002/hep.28674 27315352 PMC5090982

[17] Bano A, Chaker L, Plompen EP, et al Thyroid function and the risk of nonalcoholic fatty liver disease: the rotterdam study. J Clin Endocrinol Metab 2016;101:3204–11. 10.1210/jc.2016-1300 27270473

[18] Ting WH, Chien MN, Lo FS, et al Association of Cytotoxic T-Lymphocyte-Associated Protein 4 (CTLA4) Gene Polymorphisms with Autoimmune Thyroid Disease in Children and Adults: Case-Control Study. PLoS One 2016;11:e0154394 10.1371/journal.pone.0154394 27111218 PMC4844099

[19] Menni C, Kastenmüller G, Petersen AK, et al Metabolomic markers reveal novel pathways of ageing and early development in human populations. Int J Epidemiol 2013;42:1111–9. 10.1093/ije/dyt094 23838602 PMC3781000

[20] Qiu G, Zheng Y, Wang H, et al Plasma metabolomics identified novel metabolites associated with risk of type 2 diabetes in two prospective cohorts of Chinese adults. Int J Epidemiol 2016;45:1507–16. 10.1093/ije/dyw221 27694567

[21] An D, Oh SF, Olszak T, et al Sphingolipids from a symbiotic microbe regulate homeostasis of host intestinal natural killer T cells. Cell 2014;156:123–33. 10.1016/j.cell.2013.11.042 24439373 PMC3909465

[22] Yore MM, Syed I, Moraes-Vieira PM, et al Discovery of a class of endogenous mammalian lipids with anti-diabetic and anti-inflammatory effects. Cell 2014;159:318–32. 10.1016/j.cell.2014.09.035 25303528 PMC4260972

[23] Becker K, Frieling T, Saleh A, et al Resolution of hydatid liver cyst by spontaneous rupture into the biliary tract. J Hepatol 1997;26:1408–12. 10.1016/S0168-8278(97)80479-5 9210631

[24] Gustafson DL, Coulson AL, Feng L, et al Use of a medium-term liver focus bioassay to assess the hepatocarcinogenicity of 1,2,4,5-tetrachlorobenzene and 1,4-dichlorobenzene. Cancer Lett 1998;129:39–44. 10.1016/S0304-3835(98)00078-0 9714333

[25] Hamaguchi M, Kojima T, Itoh Y, et al The severity of ultrasonographic findings in nonalcoholic fatty liver disease reflects the metabolic syndrome and visceral fat accumulation. Am J Gastroenterol 2007;102:2708–15. 10.1111/j.1572-0241.2007.01526.x 17894848

[26] Baba M, Furuya K, Bandou H, et al Discrimination of individuals in a general population at high-risk for alcoholic and non-alcoholic fatty liver disease based on liver stiffness: a cross section study. BMC Gastroenterol 2011;11:70 10.1186/1471-230X-11-70 21669003 PMC3154156

[27] Younossi ZM, Koenig AB, Abdelatif D, et al Global epidemiology of nonalcoholic fatty liver disease-Meta-analytic assessment of prevalence, incidence, and outcomes. Hepatology 2016;64:73–84. 10.1002/hep.28431 26707365

[28] Bansal SK, Saxena V, Kandpal SD, et al The prevalence of hypertension and hypertension risk factors in a rural Indian community: A prospective door-to-door study. J Cardiovasc Dis Res 2012;3:117–23. 10.4103/0975-3583.95365 22629029 PMC3354454

[29] Boussageon R, Supper I, Bejan-Angoulvant T, et al Reappraisal of metformin efficacy in the treatment of type 2 diabetes: a meta-analysis of randomised controlled trials. PLoS Med 2012;9:e1001204 10.1371/journal.pmed.1001204 22509138 PMC3323508

[30] Nordestgaard BG, Palmer TM, Benn M, et al The effect of elevated body mass index on ischemic heart disease risk: causal estimates from a Mendelian randomisation approach. PLoS Med 2012;9:e1001212 10.1371/journal.pmed.1001212 22563304 PMC3341326

[31] Fan N, Zhang L, Xia Z, et al Sex-specific association between serum uric acid and nonalcoholic fatty liver disease in type 2 diabetic patients. J Diabetes Res 2016;2016:1–6. 10.1155/2016/3805372 PMC4921134 27382573

[32] Yang Y, Zhou Y, Cheng S, et al Effect of uric acid on mitochondrial function and oxidative stress in hepatocytes. Genet Mol Res 2016;15:15028644 10.4238/gmr.15028644 27420973

[33] Li YL, Xie H, Musha H, et al The risk factor analysis for type 2 diabetes mellitus patients with nonalcoholic fatty liver disease and positive correlation with serum uric acid. Cell Biochem Biophys 2015;72:643–7. 10.1007/s12013-014-0346-1 27352181

[34] Liu Z, Que S, Zhou L, et al Dose-response relationship of serum uric acid with metabolic syndrome and non-alcoholic fatty liver disease incidence: a meta-analysis of prospective studies. Sci Rep 2015;5:14325 10.1038/srep14325 26395162 PMC4585787

[35] Cai W, Wu X, Zhang B, et al Serum uric acid levels and non-alcoholic fatty liver disease in Uyghur and Han ethnic groups in northwestern China. Arq Bras Endocrinol Metabol 2013;57:617–22. 10.1590/S0004-27302013000800006 24343630

[36] Zhou Y, Wei F, Fan Y High serum uric acid and risk of nonalcoholic fatty liver disease: A systematic review and meta-analysis. Clin Biochem 2016;49:636–42. 10.1016/j.clinbiochem.2015.12.010 26738417

[37] Parsons WH, Kolar MJ, Kamat SS, et al AIG1 and ADTRP are atypical integral membrane hydrolases that degrade bioactive FAHFAs. Nat Chem Biol 2016;12:367–72. 10.1038/nchembio.2051 27018888 PMC4837090

[38] Kolar MJ, Kamat SS, Parsons WH, et al Branched fatty acid esters of hydroxy fatty acids are preferred substrates of the mody8 protein carboxyl ester lipase. Biochemistry 2016;55:4636–41. 10.1021/acs.biochem.6b00565 27509211 PMC5056623

[39] Moraes-Vieira PM, Saghatelian A, Kahn BB GLUT4 expression in adipocytes regulates de novo lipogenesis and levels of a novel class of lipids with antidiabetic and anti-inflammatory effects. Diabetes 2016;65:1808–15. 10.2337/db16-0221 27288004 PMC4915575

[40] Zhu QF, Yan JW, Gao Y, et al Highly sensitive determination of fatty acid esters of hydroxyl fatty acids by liquid chromatography-mass spectrometry. J Chromatogr B Analyt Technol Biomed Life Sci 2017;1061-1062:34–40. 10.1016/j.jchromb.2017.06.045 28704723

[41] Lee J, Moraes-Vieira PM, Castoldi A, et al Branched Fatty Acid Esters of Hydroxy Fatty Acids (FAHFAs) protect against colitis by regulating gut innate and adaptive immune responses. J Biol Chem 2016;291:22207–17. 10.1074/jbc.M115.703835 27573241 PMC5064000

[42] Sitthi-amorn C, Poshyachinda V Bias. Lancet 1993;342:286–8. 10.1016/0140-6736(93)91823-5 8101308

