# Supplementary material for: Risk factors and biomarkers of non-alcoholic fatty liver disease: an observational cross-sectional population survey
Source: BMJ Open. 2018 Apr 5;8(4):e019974. doi: 10.1136/bmjopen-2017-019974 (PMC5892783; doi:10.1136/bmjopen-2017-019974)
Supplement: Author's manuscript [file bmjopen-2017-019974.draft_revisions.pdf]

BMJ Open

BMJ Open is committed to open peer review. As part of this commitment we make the peer review history of every article we publish publicly available.

When an article is published we post the peer reviewers' comments and the authors' responses online. We also post the versions of the paper that were used during peer review. These are the versions that the peer review comments apply to.

The versions of the paper that follow are the versions that were submitted during the peer review process. They are not the versions of record or the final published versions. They should not be cited or distributed as the published version of this manuscript.

BMJ Open is an open access journal and the full, final, typeset and author-corrected version of record of the manuscript is available on our site with no access controls, subscription charges or pay-per-view fees (http://bmjopen.bmj.com).

If you have any questions on BMJ Open's open peer review process please email info.bmjopen@bmj.com

BMJ Open

Risk Factors and Biomarkers of Non-alcoholic Fatty Liver Disease

Journal:	*BMJ Open*
Manuscript ID	bmjopen-2017-019974
Article Type:	Research
Date Submitted by the Author:	06-Oct-2017
Complete List of Authors:	Hu, Xiao-Yu ; Wuhan Polytechnic University, School of Biology and Pharmaceutical Engineering<br>Li, Yun ; Wuhan Polytechnic University, School of Biology and Pharmaceutical Engineering<br>Li, Long-Quan ; Wuhan Polytechnic University, , School of Biology and Pharmaceutical Engineering<br>Zheng, Yuan ; Wuhan Polytechnic University, , School of Biology and Pharmaceutical Engineering<br>Lv, Jia-Hong ; Wuhan Polytechnic University, , School of Biology and Pharmaceutical Engineering<br>Huang , Shu-Chun; Wuhan Polytechnic University, School of Biology and Pharmaceutical Engineering<br>Zhao, Xiu-Ju
Keywords:	EPIDEMIOLOGY, Hepatology < INTERNAL MEDICINE, PUBLIC HEALTH

SCHOLARONE™
Manuscripts

only

Risk Factors and Biomarkers of Non-alcoholic Fatty Liver Disease

Xiao-Yu Hu[1], Yun Li[1], Long-Quan Li[1], Yuan Zheng[1], Jia-Hong Lv[1], Shu-Chun Huang[1], Xiu-Ju Zhao[1,2,*]

1School of Biology and Pharmaceutical Engineering, Wuhan Polytechnic University, 68 South Xuefu Road, Changqing Garden, Wuhan 430023, P. R. China

2Hubei Key Laboratory of Lipid Chemistry and Nutrition of Oil, Oil Crops Research Institute of the Chinese Academy of Agricultural Sciences, Wuhan 430062, P. R. China

Corresponding

Xiu-Ju Zhao

E-mail: dzrdez@163.com, Tel. and Fax: +862783956793, School of Biology and Pharmaceutical Engineering, Wuhan Polytechnic University, No.68 South Xuefu Road, Changqing Garden, Wuhan 430023, P. R. China

Abbreviations: NAFLD, non-alcoholic fatty liver disease; BMI, body mass index; BFR, body fat ratio; HBsAg, hepatitis B surface antigen; ALT, alanine aminotransferase; AST, aspartate aminotransferase

For peer review only

ABSTRACT

Objectives Non-alcoholic fatty liver disease (NAFLD) is a major public health burden in China and its prevalence is increasing. This study aimed to determine risk factors and biomarkers of NAFLD.

Design A population-based primary survey.

Setting Central China.

Participants 1500 aged over 18 and below 80 years, not currently being treated for cancer or infectious disease or no surgery in the previous year, and no previous history of cancer or an infectious disease. participants underwent clinical examination, metabolomic assay, and anthropometric assessment. Univariate and logistic regression analyses were used to assess associations between covariates and NAFLD.

Main outcome measures Risk factors and metabolic biomarkers.

Results Data from the 454 participants (mean age 44.3 ± 11.9 years) were analyzed. The prevalence of NAFLD was 24.7%. Male, body mass index ≥ 24 kg/m^2, body fat ratio (≥ 25, women; ≥ 20, men), triglycerides ≥ 1.7 mmol/L, fasting glucose ≥ 6.1 mmol/L, blood pressure ≥ 140/90 mmHg or antihypertensive drug treatment, uric acid (≥ 357 μmol/L, women; ≥ 416 μmol/L, men), and oleic acid-hydroxy oleic acid (OAHOA < 5 nmol/L) were independent predictors of NAFLD (all OR > 1, $p < 0.05$). These results were verified by the whole 1500 participants.

Conclusions NAFLD was common among the study participants. In particular, NAFLD was correlated with uric acid. We identified OAHOA as a novel marker of NAFLD prevalence. It provides a reference on the prevention of NAFLD and related metabolic diseases with the increasing urbanization, technological advancement, and population aging in China.

Strengths and limitations of this study

For peer review only

Risk factors of NAFLD in 2010.

Biomarkers of NAFLD using metabolomics.

Prediction ability of metabolic markers using ROC.

No information on education.

Keywords: uric acid; oleic acid-hydroxy oleic acid; male; non-alcoholic fatty liver disease (NAFLD)

For peer review only

INTRODUCTION

China has the world's largest population and is undergoing rapid economic growth and social reform. This advancement has paralleled demographic, lifestyle, and cultural changes that have exerted notable effects on the health profile of China's residents and placed significant constraints on the country's healthcare system. [1]

Such changes are apparent in major cities of central China, such as Wuhan. In China, the prevalence of non-alcoholic fatty liver disease (NAFLD) is increasing and it is currently the most common chronic disease among Chinese adults, with rates exceeding those of equally important epidemics including obesity, hypertension, and type II diabetes. NAFLD has been associated with an increase in overall and cardiovascular morbidity and mortality.[2-4] It has been estimated that NAFLD will be the most common indication for liver transplantation and regeneration in the coming decades.[5-7] In addition, the prevalence and impact of NAFLD in China is expected to increase as a result of population aging and the continual increase in obesity and hypertension rates. Therefore, NAFLD is a major public health burden.

The reported prevalence of NAFLD among Chinese adults ranges from 15% to 30%.[5-7] NAFLD prevalence increases with age, most notably from the fourth decade of life onward (40 - 60 years of age).[5,6,8,9] The prevalence and risk factors for NAFLD may vary across different ages and between male and female populations as a result of metabolic changes including fat redistribution and endocrine function.[10-13] However, the association of age and sex with NAFLD is still unclear. Furthermore, the association of blood pressure, obesity, dyslipidemia, insulin resistance, and diabetes with NAFLD has been widely investigated in adult cohorts

For peer review only

[14-16], and these metabolic traits are now well-accepted risk factors for NAFLD. However, limited data are available concerning the association of NAFLD with other covariant traits [17,18]. Metabolomics provides the integrated information on biological status and can investigate the molecular variations with disease phenotyping[19,20]; several markers for NAFLD have been identified previously[5,6]. Sphingolipids (SLs) and branched fatty acid esters of hydroxy fatty acids (FAHFAs) have reported to protect against autoimmune and allergic disorders and type 2 diabetes, respectively[21,22]; the effects of SLs and FAHFAs on NAFLD are largely unclear.

The aim of the present study was to investigate the prevalence of NAFLD, to elucidate the association of NAFLD with age, sex, metabolic factors, and markers of liver injury, and to identify novel metabolites associated with NAFLD risk, via clinical examination, anthropometric assessment, and targeted metabolomics.

METHODS

STUDY POPULATION

The ethics committee of Wuhan Union Hospital approved the study, and all participants provided written informed consent prior to enrolment. The Wuhan Study is a population-based cohort study of participants recruited from the Wuhan Municipality, China. Participants were prospectively recruited from routine health checkups to assess the utility of health checkups in determining the prevalence of and risk factors for NAFLD. Each participant completed a comprehensive interview and clinical examination that involved the collection of fasting blood and urine samples,

For peer review only

abdominal ultrasonography, and anthropometric assessment.

INTERVIEW

The interview, which preceded the clinical examination, was conducted by a physician and was designed to obtain information concerning demographic characteristics, medical history, and comorbid conditions. Participants aged over 18 and below 80 years were included if they were not currently being treated for cancer or infectious disease or had undergone surgery in the previous year, and if they had no previous history of cancer or an infectious disease.

BIOCHEMISTRY

Fasting blood and urine samples were collected on the morning of the clinical examination. Blood triglycerides, glucose, alanine aminotransferase (ALT), aspartate aminotransferase (AST), gamma-glutamyl transferase, alkaline phosphatase, total bilirubin, and urine uric acid levels were measured using automatic enzymatic procedures (Nanjing Jiancheng Sci-tech, China). Hepatitis B surface antigen (HBsAg), triiodothyronine, thyroxine (T4), and thyroid-stimulating hormone were measured via automatic immunoassays (Roche Diagnostics GmbH, Mannheim, DE).

DIAGNOSIS AND SUBTYPES OF LIVER DISEASES

Abdominal ultrasonography was performed on all participants by accredited technicians using a Hitachi HI VISION 900 ultrasound machine. Images were stored digitally and evaluated by a senior pathologist. LD subtypes were diagnosed by the technician according to published protocols [23-25]. Individuals with any of the

For peer review only

fol-lowing possible secondary causes of fatty liver were excluded from the analyses: (1) excessive alcohol consumption, (2) positive HBsAg or anti-HCV, and (3) use of pharmacological agents historically associated with fatty liver (i.e., amiodarone, corticosteroids, methotrexate, and tamoxifen).[13]

METABOLIC COVARIATES

Anthropometric measurements were performed by trained nurses. Body mass index (BMI) was calculated as measured weight (kg) divided by height squared (m^2). Body fat ratio (BFR) was defined as fat weight divided by body weight. The average of two blood pressure measurements, obtained at a single visit in the sitting position after a 10-min rest, was used for analysis.

Metabolic traits were defined: obesity (BMI ≥ 24 kg/m^2), hypertension (blood pressure ≥ 140/90 mmHg or antihypertensive drug treatment), BFR ≥ 25 for women or ≥ 20 for men, blood triglycerides ≥ 1.7 mmol/L, blood fasting glucose ≥ 6.1 mmol/L, liver enzyme elevation (AST ≥ 40 U/L or ALT ≥ 40 U/L), uric acid ≥ 357 μmol/L for women or ≥ 416 μmol/L for men, or impaired OAHOA(< 5 nmol/L).

TARGETED LC/MS ANALYSIS OF SLS AND FAHFAS

To identify SLs[21], silica column-affectionate lipid fractions were analyzed by reverse-phase HPLC-MS/MS (Agilent C18 column connected with Thermo Scientific LTQXL; gradients of water to methanol, 10:90 to 0:100) to identify spots present in both control and LD. Identified lipid species of interest were purified by reverse-phase HPLC (Varian Prostar/Agilent C18 column) and subjected to structural analysis.

For peer review only

FAHFAs were measured by HPLC-MS [22] (an Agilent 6410 Triple Quad LC/MS via MRM in negative ionization mode). Briefly, Extracted and fractionated samples were reconstituted in 25 ml MeOH; 10 ml was injected for analysis. A Luna C18(2) (Phenomenex) column was used with an in-line filter (Phenomenex). Distinct FAHFAs were resolved via isocratic flow (0.2 ml/min for 120 min, solvent: 93:7 MeOH:H_2O with 5 mM ammonium acetate and 0.01% ammonium hydroxide). Transitions for endogenous OAHOAs were m/z 561.5 → m/z 281.2 (Collision Energy [CE] = 30 V), and m/z 561.5 → m/z 279.2 (CE = 25 V).

STATISTICAL ANALYSIS

Baseline analyses were performed using descriptive statistics. U-tests, chi-square tests, or Wilcoxon rank-sum tests (for medians) and Student's t-tests (for means) were used to assess the significance of differences in the distribution of categorical data and continuous data, respectively. To examine associations between traits and NAFLD, we performed binary or multiple logistic regression analyses. We calculated the area under the receiver operating curve (AUC) to assess prediction ability of metabolic markers. A p-value of < 0.05 was considered statistically significant unless stated otherwise. Statistical analyses were performed using SPSS software version 17.0 (SPSS Inc., Chicago, IL, USA).

RESULTS

STUDY POPULATION

In total, 1500 participants were enrolled. Twenty-one participants were excluded due to the absence of clinical data. Thus, a total of 1479 study participants were

For peer review only

included in the final analysis, of which 447 reported their age and had no more than one disease, if any (denoted as the "subset"). Patients' general characteristics are shown in Table 1. Men accounted for 40% of the study population (38% of the subset). Participants' mean age was 44.3 ± 11.9 (range 20–74) years.

PREVALENCE OF NAFLD

The prevalence of NAFLD, as determined by ultrasound and biopsy, was 24.7%, (22.8% in the subset).

The peak prevalence of NAFLD in this study was 26.4% and 26.3% between the ages of 30–40 years and 50–60 years, respectively (Table 2).

The prevalence of NAFLD demonstrated an increasing trend with advancing age (OR 1.049, $p = 0.607$), and after adjustment for sex and metabolic features, NAFLD tended to be inversely related to age (OR 0.844; 95% CI 0.667–1.068; $p = 0.157$). NAFLD was diagnosed in 37.4% of men and 13.8% of women ($p = 0.084$). In multivariate analysis, after adjustment for metabolic features and age, male sex was associated with NAFLD (OR 0.287; 95% CI 0.167–0.493; $p < 0.001$, Table 3).

ASSOCIATION BETWEEN NAFLD AND METABOLIC FEATURES

Among metabolic covariates, obesity and hypertension occurred more frequently in participants with advancing age (OR 1.471 and 1.822, respectively; $p < 0.001$). Increased body fat was more prevalent in women than in men (OR 2.042; p < 0.001; [OR 1.754; p = 0.007 in the subset]), and obesity (OR 0.537, $p < 0.001$), increased blood lipids (OR 0.829, p < 0.101), and hypertension (OR 0.769; p < 0.041) were more prevalent in men than in women (OR 0.472, p <0.001; OR 0.902, p < 0.673; and

For peer review only

OR 0.602; $p < 0.080$, respectively, in the subset).

In univariate analysis, all metabolic and anthropometric traits were significantly associated with NAFLD. In logistic regression analysis, after adjustment for sex, BMI ≥ 24 kg/m^2 (OR 8.494; 95% CI 5.581–12.928; $p < 0.001$), BFR ≥ 25 for women and ≥ 20 for men (OR 1.833; 95% CI 1.286–2.756; $p = 0.001$), triglycerides ≥ 1.7 mmol/L (OR 1.340; 95% CI 1.006–1.785; $p = 0.046$), fasting glucose ≥ 6.1 mmol/L (OR 3.324; 95% CI 1.888–5.850; $p < 0.001$), blood pressure (BP)≥ 140/90 mmHg or antihypertensive drug treatment (OR 1.451, 95% CI 1.069–1.970; $p = 0.017$), uric acid ≥ 357 μmol/L for women and ≥ 416 μmol/L for men (OR 1.448, 95% CI 0.869–2.412; $p = 0.156$), and total OAHOA < 5 nmol/L (OR 1.340, 95% CI 1.006-1.785; $p = 0.046$) [after adjustment for age stages and sex in the subset, OR for BMI, BFR, and BP: 11.738 (95% CI 5.193–26.530; $p < 0.001$), 2.285 (95% CI 1.067–4.893; $p < 0.033$) and 1.865 (95% CI 0.910–3.824; $p < 0.089$), respectively] were independent predictors of NAFLD (Table 3 and Table 4). The prevalence of NAFLD demonstrated an increasing trend with lowering sphingosines (OR 1.448, $p = 0.156$; Table 4). Substitution of age ranges for continuous data (i.e., absolute age) in logistic regression did not modify the relationships. When each metabolic trait was analyzed independently, after adjustment for age and sex, ORs increased for obesity and hypertension with increasing age, and ORs increased for BFR with increasing age for patients aged <50 years and decreased for patients aged ≥50 years. However, significant interactions were only observed between age and obesity ($p < 0.001$), and between age and hypertension ($p < 0.001$).

ASSOCIATION BETWEEN NAFLD AND LIVER ENZYMES

For peer review only

Participants with NAFLD had higher liver enzyme levels than participants without this condition ($p < 0.001$). Abnormal liver enzymes were also significantly associated with NAFLD, independent of sex (OR 3.150, $p < 0.001$). Normal liver enzyme levels, defined according to local guidelines (ALT <40 U/L), were found in 85% of participants with NAFLD.

DISCUSSION

For peer review only

Our results demonstrated that NAFLD was strong associated with metabolic traits including body fat, obesity, hyperlipidemia, and impaired fasting glucose. We observed a higher prevalence of NAFLD in men than in women. We conformed the association of impaired uric acid, and abnormal liver enzymes with NAFLD; and further identified impaired total OAHOA as a novel biomarker of NAFLD incidence.

The average and peak prevalence of NAFLD in this study was 24.7 and more than 26%, respectively. The peak average and prevalence of NAFLD in Shanghai [5] was 20.82% and 28.44%, respectively, and those in Guangdong [6] was 17.2% and 27.4%, respectively. The former study was conducted from 2002 to 2003, and the latter in 2005. Our study, performed in 2010, revealed that the average prevalence is increasing, and the peak prevalence occurred at a younger age than was reported by the previous studies in Shanghai (age of 60-69) [5] and Guangdong (age of 60-69) [6]. Thus, more attention should be given to the health of people in their 30s, when many marry and adopt lifestyle changes, especially in urban China. Unfortunately, we did not have access to information on participants' activity levels, and thus, the effects of

physical activity on NAFLD were not assessed. The reasons underlying the observed earlier peak prevalence require further investigation.

We observed that male sex was a risk factor for NAFLD. This finding differs from the results of the Guangdong study [6], and the Rotterdam study [13]. However, this finding is similar to the results of the Shanghai study.

There are several possibilities for this discrepancy. First, the observed sex difference in NAFLD prevalence may be the result of body fat, which is a risk factor for NAFLD and was higher in men than in women in this study. Therefore, male sex may promote NAFLD through mediators such as increased body fat and hormonal change. Second, the sex difference in NAFLD may also result from a lower prevalence of elevated serum triglycerides, glucose, and blood pressure in females compared with males. A lower prevalence of elevated serum triglycerides, glucose and blood pressure in females was reported in a previous study [26]. However, a causal relationship between NAFLD and these serum metabolic markers, as suggested by previous research [27,28], could not be established by our study, given its cross-sectional design. Third, abnormal liver enzymes were more prevalent in men than in women, and both were independent predictors of NAFLD.

We observed that the associations of the identified metabolites with NAFLD risk were pronounced in the Wuhan cohort. These metabolites might play an important role in the development of onset NAFLD. Uric acid was specifically associated with NAFLD in men with type 2 diabetes, independent of insulin resistance and other metabolic factors [29]. High concentrations of uric acid induce the accumulation of

For peer review only

reactive oxygen species in hepatocyte mitochondria, ultimately leading to mitochondrial damage [30], and uric acid was positively correlated with other metabolic traits and was a risk factor for type 2 diabetes mellitus with NAFLD [31].

Serum uric acid (SUA) is the end-product of purine nucleotide catabolism.[32] The SUA subjects have an approximately 2-fold higher risk of NAFLD as defined by ultrasonogra-phy. The risk may be independent of age, gender, and obesity (as estimated by BMI and waist circumference). Among subjects with in-creased SUA levels, women likely showed a greater risk of NAFLD than men.[33]

OAHOAs are present in humans, which are an endogenous branched fatty acid esters of hydroxy fatty acids (FAHFAs) and levels are reduced with obesity and insulin resistance. OAHOA levels in serum correlate highly with whole-body insulin sensitivity. OAHOAs are endogenous GPR120 ligands and may also exert anti-inflammatory effects in vivo through lipid-activated GPCRs, such as GPR120. Changes in the levels of these anti-inflammatory metabolites and in their signaling pathways may provide new targets for metabolic and inflammatory diseases [22].

Our study also has some potential limitations. First, we cannot rule out the possibility of biased selection, since non-responders may have had different morbidities. Volunteers in research studies tend to be better educated, healthier, and have better lifestyles [34]. Consequently, estimations of the prevalence of NAFLD may have been biased. Second, only 454 of the 1500 participants provided information on their ages, and we did not obtain data on participants' educational levels or incomes. Third, specific OAHOA isomers [22] require further study.

For peer review only

CONCLUSIONS

NAFLD is common in the study population. In this cohort-based primary survey, we found that NAFLD was associated with male sex. In particular, NAFLD was correlated with uric acid. Moreover, we identified OAHOA as a novel biomarker of NAFLD. Further studies are needed to explore the potential factors contributing to these relationships. However, this study is valuable in that it provides a reference on the prevention of NAFLD and related metabolic diseases with the increasing urbanization, technological advancement, and population aging in China.

Contributors Designed the study: Xiu-Ju Zhao. Analyzed data: Xiao-Yu Hu, Yun Li, Long-Quan Li, Yuan Zheng, Jia-Hong Lv, Shu-Chun Huang, Xiu-Ju Zhao. Wrote and critically review the manuscript: Xiao-Yu Hu, Xiu-Ju Zhao.

Ethics approval The ethics committee of Wuhan Union Hospital approved the study, and all participants provided written informed consent prior to enrolment.

Data sharing statement Data are available on request.

Conflict of interest: There is no conflict of interest.

FUNDING STATEMENT: This work was supported in part by the Opening Project of Hubei Key Laboratory of Lipid Chemistry and Nutrition of Oil (201506), and National Nature Science Foundation of China (21602166). They had no role in this study.

For peer review only

REFERENCES

1. Yusuf S, Reddy S, Ounpuu S, Anand S. Global burden of cardiovascular diseases: part I: general considerations, the epidemiologic transition, risk factors, and impact of urbanization. *Circulation* 2001;**104**: 2746-2753.
2. Dunn W, Xu R, Wingard D, Rogers C, Angulo P, Younossi Z *et al*. Suspected Nonalcoholic Fatty Liver Disease and Mortality Risk in a Population-Based Cohort Study. *Am J Gastroenterol* 2008;**103**: 2263-2271.
3. Ong JP, Pitts A, Younossi ZM. Increased overall mortality and liver-related mortality in non-alcoholic fatty liver disease. *J Hepatol* 2008;**49**: 608-612.
4. Targher G., Day CP, Bonora E. Risk of cardiovascular disease in patients with nonalcoholic fatty liver disease. *N Engl J Med* 2010;**36**: 1341-1350.
5. Fan JG, Zhu J, Li X J, Chen L, Li L, Dai F *et al*. Prevalence of and risk factors for fatty liver in a general population of Shanghai, China. *J Hepatol* 2005;**43**: 508-514.
6. Zhou YJ, Li YY, Nie YQ, Ma JX, Lu LG, Shi SL *et al*. Prevalence of fatty liver disease and its risk factors in the population of South China. *World J Gastroenterol* 2007;**13**: 6419-6424.
7. Fan JG., Farrell GC. Epidemiology of non-alcoholic fatty liver disease in China. *J Hepatol* 2009;**50**: 204-210.
8. Schwimmer JB, Deutsch R, Kahen T, Lavine J E, Stanley C, Behling C. Prevalence of fatty liver in children and adolescents. *Pediatrics* 2006;**118**: 1388-1393.
9. Ayonrinde OT, Olynyk JK, Beilin LJ, Mori TA, Pennell CE, de Klerk N *et al*. Gender-specific differences in adipose distribution and adipocytokines influence adolescent nonalcoholic fatty liver disease. *Hepatology* 2011;**53**: 800-809.
10. Roy AK, Chatterjee B. Sexual Dimorphism in the Liver. *Annu Rev Physiol* 1983;**45**: 37-50.
11. Sepe A, Tchkonia T, Thomou T, Zamboni M, Kirkland JL. Aging and regional differences in fat cell progenitors - a mini-review. *Gerontology* 2011;**57**: 66-75.
12. Takasugi M, Hayakawa K, Arai D, Shiota K. Age- and sex-dependent DNA hypomethylation controlled by growth hormone in mouse liver. *Mech Ageing Dev* 2013;**134**: 331-337.
13. Koehler EM, Schouten JN, Hansen BE, van Rooij FJ, Hofman A, Stricker BH, *et al*. Prevalence and risk factors of non-alcoholic fatty liver disease in the elderly: results from the Rotterdam study. *J Hepatol* 2012;**57**: 1305-1311.
14. Marchesini G, Brizi M, Morselli-Labate A M, Bianchi G, Bugianesi E, McCullough AJ *et al*. Association of nonalcoholic fatty liver disease with insulin resistance. *Am J Med* 1999;**107**: 450-455.
15. Pagano G, Pacini G, Musso G, Gambino R, Mecca F, Depetris N *et al*. Nonalcoholic steatohepatitis, insulin resistance, and metabolic syndrome: further evidence for an etiologic association. *Hepatology* 2002;**35**: 367-372.
16. Cui J, Chen CH, Lo MT, Schork N, Bettencourt R, Gonzalez MP *et al*. Shared genetic effects between hepatic steatosis and fibrosis: A prospective twin study. *Hepatology* 2016; **64**: 1547–1558.
17. Bano A, Chaker L, Plompen EPC, Hofman A, Dehghan A, Franco OH *et al*. Thyroid function and the risk of non-alcoholic fatty liver disease: The Rotterdam Study. *J Clin Endocrinol Metab*. 2016; **101**: 3204–3211.
18. Ting WH, Chien MN, Lo FS, Wang CH, Huang CY, Lin CL *et al*. Association of cytotoxic

For peer review only

T-lymphocyte-associated protein 4 (CTLA4) gene polymorphisms with autoimmune thyroid disease in children and adults: case-control study. *PLoS One*. 2016; **11**:e0154394.

19. Menni C, Kastenmüller G, Petersen AK, Bell JT, Psatha M, Tsai PC *et al*. Metabolomic markers reveal novel pathways of ageing and early development in human populations. *Int J Epidemiol* 2013; **42**: 1111–1119.

20. Qiu, G. et al. Plasma metabolomics identified novel metabolites associated with risk of type 2 diabetes in two prospective cohorts of Chinese adults. Int J Epidemiol.2016; 45: 1507-1516.

21. An D, Oh SF, Olszak T, Neves JF, Avci FY, Erturk-Hasdemir D *et al*. Sphingolipids from a symbiotic microbe regulate homeostasis of host intestinal natural killer T cells. *Cell* 2014;**156**: 123-133.

22. Yore MM, Syed I, Moraes-Vieira PM, Zhang T, Herman MA, Homan EA *et al*. Discovery of a class of endogenous mammalian lipids with anti-diabetic and anti-inflammatory effects. *Cell* 2014;**159**: 318-332.

23. Becker K, Frieling T, Saleh A, Haussinger D. Resolution of hydatid liver cyst by spontaneous rupture into the biliary tract. *J Hepatol* 1997;**26**: 1408-1412.

24. Gustafson DL, Coulson AL, Feng L, Pott WA, Thomas RS, Chubb LS *et al*. Use of a medium-term liver focus bioassay to assess the hepatocarcinogenicity of 1,2,4,5-tetrachlorobenzene and 1,4-dichlorobenzene. *Cancer Lett* 1998; **129**: 39-44.

25. Hamaguchi M, Kojima T, Itoh Y, Harano Y, Fujii K, Nakajima T *et al*. The severity of ultrasonographic findings in nonalcoholic fatty liver disease reflects the metabolic syndrome and visceral fat accumulation. *Am J Gastroenterol* 2007; **102**: 2708-2715.

26. Bansal SK, Saxena V, Kandpal SuD, Gray WK, Walker RW, Goel D. The prevalence of hypertension and hypertension risk factors in a rural Indian community: A prospective door-to-door study. *J Cardiovasc Dis Res* 2012;**3**: 117-123.

27. Boussageon R, Supper I, Bejan-Angoulvant T, Kellou N, Cucherat M, Boissel JP *et al*. Reappraisal of metformin efficacy in the treatment of type 2 diabetes: a meta-analysis of randomised controlled trials. *PLoS Med* 2012; **9**:e1001204.

28. Nordestgaard BG, Palmer TM, Benn M, Zacho J, Tybjærg-Hansen A, Davey Smith G *et al*. The effect of elevated body mass index on ischemic heart disease risk: causal estimates from a mendelian randomisation approach. *PLoS Med* 2012; **9**:e1001212.

29. Fan N, Zhang L, Xia Z, Peng L, Wang Y, Peng Y. Sex-specific association between serum uric acid and nonalcoholic fatty liver disease in type 2 diabetic patients. *J Diabetes Res* 2016; **2016**: 3805372.

30. Yang Y, Zhou Y, Cheng S, Sun JL, Yao H, Ma L. Effect of uric acid on mitochondrial function and oxidative stress in hepatocytes. *Genet Mol Res* 2016; **15**: 15028644.

31. Li YL, Xie H, Musha H, Xing Y, Mei CX, Wang HJ *et al*. The risk factor analysis for type 2 diabetes mellitus patients with nonalcoholic fatty liver disease and positive correlation with serum uric acid. *Cell Biochem Biophys* 2015; **72**: 643-647.

32. Liu Z, Que S, Zhou L, Zheng S. Dose-response relationship of serum uric acid with metabolic syndrome and non-alcoholic fatty liver disease incidence: a meta-analysis of prospective studies. *Sci Rep* 2015; **5**: 14325

33. Zhou Y, Wei F, Fan Y. High serum uric acid and risk of nonalcoholic fatty liver disease: A systematic review and meta-analysis. *Clin Biochem* 2016; **49**: 636 ‑ 642.

34. Sitthi-amorn C, Poshyachinda V. Bias. *Lancet*. 1993;**342**: 286-288

For peer review only

Table 1 Participant characteristics.

a. Subset

Indicators	Total	NAFLD	No NAFLD	P value
**n**	447	102 (22.8%)	345 (77.2%)	
**Age (years)**	44.3 ±11.9	44.6 ±10.8	44.0 ±12.1	0.609
**Age stage**	4.0±1.2	4.0±1.2	3.9±1.2	0.608
**Obesity**	235 (51.8%)	94	141	<0.001
**Body fat**	309 (68.1%)	89	220	<0.001
**Blood lipids**	87 (19.2%)	21	66	0.745
**Hypertension**	55 (12.1%)	23	32	0.003
**Male**	171 (38.3%)	64	107	<0.001
**Elevated liver enzymes**	18 (4.0%)	7	11	0.174

Age stages of 20-30, 30-40, 40-50, 50-60, ≥60 are 2, 3, 4, 5, 6.

b Total study population

Indicators	Total	NAFLD	No NAFLD	P value
**n**	1479	365 (24.7%)	1114 (75.3%)	
**Obesity**	809 (54.7%)	334	475	<0.001
**Body fat**	1003 (67.8%)	309	694	<0.001

For peer review only

**Blood lipid**	458 (30.9%)	163	295	<0.001
**High blood glucose**	71 (4.8%)	43	28	<0.001
**Hypertension**	314 (21.2%)	129	185	<0.001
**Abnormal uric acid**	83 (5.6%)	38	45	<0.001
**Male**	590 (39.8%)	214	376	<0.001
**Impaired liver function**	32 (0.2%)	12	20	0.142
**Elevated liver enzymes**	110 (0.7%)	53	57	<0.001

Conditions were defined as follows: obesity (BMI ≥ 24 kg/m^2), hypertension (blood pressure ≥ 140/90 mmHg or antihypertensive drug treatment), Body fat ratio ≥ 25 for women or ≥ 20 for men, blood triglycerides ≥ 1.7 mmol/L, blood fasting glucose ≥ 5.6 mmol/L, impaired liver function (positive HBsAg), liver enzyme elevation (AST ≥ 40 U/L or ALT ≥ 40 U/L), uric acid ≥ 357 μmol/L for women or ≥ 416 μmol/L for men.

Mean values are provided with standard deviation, unless otherwise noted as n (%). Differences between participants with and without NAFLD were evaluated with t-tests or the Wilcoxon-Mann-Whitney test for continuous variables and the chi-squared test for categorical variables. NAFLD, non-alcoholic fatty liver disease.

For peer review only

Table 2. NAFLD prevalence across age range.

**Age range (years)**	**Participants, n**	**Participants with NAFLD, n**	**NAFLD prevalence,%**	**U-test**
20-30	64	10	15.6	1.15
30-40	102	27	26.4	0.85
40-50	121	26	21.5	0.13
50-60	114	30	26.3	1.00
≥60	53	9	17.0	0.78
Total	454	102	22.4	1.64 (p=0.1)

NAFLD, non-alcoholic fatty liver disease.

For peer review only

Table 3. Multivariate adjusted models for liver disease subtypes.

a Subset

Variables	OR	*p* value
Sex (female)	0.267	<0.001
Obesity	16.667	<0.001
Body fat	3.859	<0.001
Elevated liver enzyme	2.237	0.105
Hypertension	2.848	0.001

b The whole

Variables	OR	*p* value
Sex (female)	0.359	<0.001
Obesity	14.109	<0.001
Body fat	3.292	<0.001
Blood lipid	2.240	<0.001
Blood glucose	5.185	<0.001
Impaired liver function	1.859	0.094
Elevated liver enzyme	3.150	<0.001
Hypertension	2.739	<0.001

Conditions were defined as follows: female sex=1, obesity (BMI ≥ 24 kg/m^2), hypertension (blood pressure ≥ 140/90 mmHg or antihypertensive drug treatment), body fat ratio ≥ 25 for women or ≥ 20 for men, blood fasting

For peer review only

glucose ≥ 6.1 mmol/L, liver enzyme elevation (AST ≥ 40 U/L or ALT ≥ 40 U/L)

NAFLD, non-alcoholic fatty liver disease.

Odds ratios and the corresponding p values derived from binary or multiple logistic regression analyses using SPSS software version 17.0 (SPSS Inc., Chicago, IL, USA)

For peer review only

Table 4. Metabolites associated with non-alcoholic fatty liver disease.

Metabolite	OR, P value	AUC, P value
**Uric acid**	2.755 <0.001	0.579 <0.001
**Sphingosine**	1.448 0.156	0.489 0.760
**OAHOA**	1.340 0.046	0.612 0.001

Conditions were defined: Sphingosine < 2 nmol/L, OAHOA (oleic acid -hydroxy oleic acid) < 5 nmol/L.

NAFLD, non-alcoholic fatty liver disease.

Odds ratios (OR), area under curve (AUC) and the corresponding p values derived from binary or multiple logistic regression, or ROC analyses using SPSS software version 17.0 (SPSS Inc., Chicago, IL, USA)

For peer review only

CONSORT 2010 checklist of information to include when reporting a randomised trial*

Section/Topic	Item No	Checklist item	Reported on page No
**Title and abstract**			
	1a	Identification as a randomised trial in the title	1
	1b	Structured summary of trial design, methods, results, and conclusions (for specific guidance see CONSORT for abstracts)	3
**Introduction**			
Background and objectives	2a	Scientific background and explanation of rationale	5-6
	2b	Specific objectives or hypotheses	5-6
**Methods**			
Trial design	3a	Description of trial design (such as parallel, factorial) including allocation ratio	6
	3b	Important changes to methods after trial commencement (such as eligibility criteria), with reasons	6
Participants	4a	Eligibility criteria for participants	6
	4b	Settings and locations where the data were collected	6
Interventions	5	The interventions for each group with sufficient details to allow replication, including how and when they were actually administered	7
Outcomes	6a	Completely defined pre-specified primary and secondary outcome measures, including how and when they were assessed	7
	6b	Any changes to trial outcomes after the trial commenced, with reasons	7
Sample size	7a	How sample size was determined	6
	7b	When applicable, explanation of any interim analyses and stopping guidelines	6
Randomisation:			
Sequence generation	8a	Method used to generate the random allocation sequence	7
	8b	Type of randomisation; details of any restriction (such as blocking and block size)	7
Allocation concealment mechanism	9	Mechanism used to implement the random allocation sequence (such as sequentially numbered containers), describing any steps taken to conceal the sequence until interventions were assigned	7
Implementation	10	Who generated the random allocation sequence, who enrolled participants, and who assigned participants to interventions	7
Blinding	11a	If done, who was blinded after assignment to interventions (for example, participants, care providers, those	-

For peer review only

For peer review only - http://bmjopen.bmj.com/site/about/guidelines.xhtml

		assessing outcomes) and how	
	11b	If relevant, description of the similarity of interventions	-
Statistical methods	12a	Statistical methods used to compare groups for primary and secondary outcomes	8
	12b	Methods for additional analyses, such as subgroup analyses and adjusted analyses	8
**Results**			
Participant flow (a diagram is strongly recommended)	13a	For each group, the numbers of participants who were randomly assigned, received intended treatment, and were analysed for the primary outcome	7
	13b	For each group, losses and exclusions after randomisation, together with reasons	7
Recruitment	14a	Dates defining the periods of recruitment and follow-up	7
	14b	Why the trial ended or was stopped	7
Baseline data	15	A table showing baseline demographic and clinical characteristics for each group	7
Numbers analysed	16	For each group, number of participants (denominator) included in each analysis and whether the analysis was by original assigned groups	7
Outcomes and estimation	17a	For each primary and secondary outcome, results for each group, and the estimated effect size and its precision (such as 95% confidence interval)	7
	17b	For binary outcomes, presentation of both absolute and relative effect sizes is recommended	7
Ancillary analyses	18	Results of any other analyses performed, including subgroup analyses and adjusted analyses, distinguishing pre-specified from exploratory	8
Harms	19	All important harms or unintended effects in each group (for specific guidance see CONSORT for harms)	7
**Discussion**			
Limitations	20	Trial limitations, addressing sources of potential bias, imprecision, and, if relevant, multiplicity of analyses	18
Generalisability	21	Generalisability (external validity, applicability) of the trial findings	7
Interpretation	22	Interpretation consistent with results, balancing benefits and harms, and considering other relevant evidence	18
**Other information**			
Registration	23	Registration number and name of trial registry	-
Protocol	24	Where the full trial protocol can be accessed, if available	-
Funding	25	Sources of funding and other support (such as supply of drugs), role of funders	2

*We strongly recommend reading this statement in conjunction with the CONSORT 2010 Explanation and Elaboration for important clarifications on all the items. If relevant, we also recommend reading CONSORT extensions for cluster randomised trials, non-inferiority and equivalence trials, non-pharmacological treatments, herbal interventions, and pragmatic trials. Additional extensions are forthcoming: for those and for up to date references relevant to this checklist, see www.consort-statement.org.

For peer review only - http://bmjopen.bmj.com/site/about/guidelines.xhtml

Risk Factors and Biomarkers of Non-alcoholic Fatty Liver Disease: a large-scale observational cross-sectional population survey

Journal:	*BMJ Open*
Manuscript ID	bmjopen-2017-019974.R1
Article Type:	Research
Date Submitted by the Author:	27-Dec-2017
Complete List of Authors:	Hu, Xiao-Yu ; Wuhan Polytechnic University, School of Biology and Pharmaceutical Engineering<br>Li, Yun ; Wuhan Polytechnic University, School of Biology and Pharmaceutical Engineering<br>Li, Long-Quan ; Wuhan Polytechnic University, , School of Biology and Pharmaceutical Engineering<br>Zheng, Yuan ; Wuhan Polytechnic University, , School of Biology and Pharmaceutical Engineering<br>Lv, Jia-Hong ; Wuhan Polytechnic University, , School of Biology and Pharmaceutical Engineering<br>Huang , Shu-Chun; Wuhan Polytechnic University, School of Biology and Pharmaceutical Engineering<br>Zhang, Wei-Nong ; Wuhan Polytechnic University, School of Biology and Pharmaceutical Engineering<br>Liu, Liang; Wuhan Polytechnic University, School of Biology and Pharmaceutical Engineering<br>Zhao, Ling; Wuhan Polytechnic University, School of Biology and Pharmaceutical Engineering<br>Liu, Zhiguo; Wuhan Polytechnic University, School of Biology and Pharmaceutical Engineering<br>Zhao, Xiu-Ju
**Primary Subject Heading**:	Public health
Secondary Subject Heading:	Gastroenterology and hepatology, Epidemiology
Keywords:	EPIDEMIOLOGY, Hepatology < INTERNAL MEDICINE, PUBLIC HEALTH

Risk Factors and Biomarkers of Non-alcoholic Fatty Liver Disease: a large-scale observational cross-sectional population survey

Xiao-Yu Hu[1], Yun Li[1], Long-Quan Li[1], Yuan Zheng[1], Jia-Hong Lv[1], Shu-Chun Huang[1], Weinong Zhang[1], Liang Liu[1], Ling Zhao[1], Zhuiguo Liu[1], Xiu-Ju Zhao[1,2,3*]

1 School of Biology and Pharmaceutical Engineering, Wuhan Polytechnic University, 68 South Xuefu Road, Changqing Garden, Wuhan, Hubei 430023, P. R. China

2 Hubei Key Laboratory of Lipid Chemistry and Nutrition of Oil, Oil Crops Research Institute of the Chinese Academy of Agricultural Sciences, Wuhan, Hubei 430062, P. R. China

3 Department of Nutrition and Food Science, Texas A&M University, College Station, TX 77843, U.S.A.

Corresponding

Xiu-Ju Zhao

E-mail: dzrdez@163.com, Tel. and Fax: +862783956793, School of Biology and Pharmaceutical Engineering, Wuhan Polytechnic University, 68 South Xuefu Road, Changqing Garden, Wuhan 430023, P. R. China

For peer review only

Abbreviations: NAFLD, non-alcoholic fatty liver disease; BMI, body mass index; BFR, body fat ratio; HBsAg, hepatitis B surface antigen; ALT, alanine aminotransferase; AST, aspartate aminotransferase

ABSTRACT

Objectives Non-alcoholic fatty liver disease (NAFLD) is a major public health burden in China and its prevalence is increasing. This study aimed to determine risk factors and biomarkers of NAFLD.

Design A population-based observational cross-sectional primary survey.

Setting Central China.

Participants 1500 aged over 18 and below 80 years, not currently being treated for cancer or infectious disease or no surgery in the previous year, and no previous history of cancer or an infectious disease. Participants underwent clinical examination, metabolomic assay, and anthropometric assessment. Univariate and logistic regression analyses were used to assess associations between covariates and NAFLD.

Main outcome measures Risk factors and metabolic biomarkers.

Results Data from the 454 participants (mean age 44.3 ± 11.9 years) were analyzed. The prevalence of NAFLD was 24.7%. Male, body mass index ≥ 24 kg/m^2, body fat ratio (≥ 25, women; ≥ 20, men), triglycerides ≥ 1.7 mmol/L, fasting glucose ≥ 6.1 mmol/L, blood pressure ≥ 140/90 mmHg or antihypertensive drug treatment, uric acid (≥ 357 μmol/L, women; ≥ 416 μmol/L, men), and oleic acid-hydroxy oleic acid (OAHOA < 5 nmol/L) were independent predictors of NAFLD (all OR > 1, $p < 0.05$). These results were verified by the whole 1500 participants.

Conclusions NAFLD was common among the study participants. In particular, NAFLD was correlated with uric acid. We identified OAHOA as a novel marker of NAFLD prevalence. It provides a reference on the prevention of NAFLD and related metabolic diseases with the rapid urbanization, technological advancement, and population aging in China over the past recent decades.

Strengths and limitations of this study

For peer review only

Risk factors of NAFLD in central China.

Biomarkers of NAFLD using metabolomics.

Prediction ability of metabolic markers using ROC.

No information on education.

Keywords: uric acid; oleic acid-hydroxy oleic acid; male; non-alcoholic fatty liver disease (NAFLD)

For peer review only

INTRODUCTION

China has the world's largest population and is undergoing rapid economic growth and social reform. This advancement has paralleled demographic, lifestyle, and cultural changes that have exerted notable effects on the health profile of China's residents and placed significant constraints on the country's healthcare system. [1]

Such changes are apparent in major cities of central China, such as Wuhan. In China, the prevalence of non-alcoholic fatty liver disease (NAFLD) is increasing and it is currently the most common chronic disease among Chinese adults, with rates exceeding those of equally important epidemics including obesity, hypertension, and type II diabetes. NAFLD has been associated with an increase in overall and cardiovascular morbidity and mortality.[2-4] It has been estimated that NAFLD will be the most common indicator for liver transplantation and regeneration in the coming decades.[5-7] In addition, the prevalence and impact of NAFLD in China is expected to increase as a result of population aging and the continual increase in obesity and hypertension rates with reforming and open-up over the recent decades. Therefore, NAFLD is a major public health burden.

The reported prevalence of NAFLD among Chinese adults ranges from 15% to 30%.[5-7] NAFLD prevalence increases with age, most notably from the fourth decade of life onward (40 - 60 years of age).[5,6,8,9] The prevalence and risk factors for NAFLD may vary across different ages and between male and female populations as a result of metabolic changes including fat redistribution and endocrine function.[10-13] However, the association of age and sex with NAFLD in central China is still unclear. Furthermore, the association of hypertension, obesity,

For peer review only

hyperlipidemia, insulin resistance, and diabetes with NAFLD has been widely investigated in adult cohorts [14-16], and these metabolic traits are now well-accepted risk factors for NAFLD. However, limited data are available concerning the association of NAFLD with other covariant traits [17,18]. Metabolomics provides the integrated information on biological status and can investigate the molecular variations with disease phenotyping[19,20]; several markers for NAFLD have been identified previously[5,6]. Sphingolipids (SLs) and branched fatty acid esters of hydroxy fatty acids (FAHFAs) have reported to protect against autoimmune and allergic disorders and type 2 diabetes, respectively[21,22]; the effects of SLs and FAHFAs on NAFLD are largely unclear.

The aim of the present study was to investigate the prevalence of NAFLD, to elucidate the association of NAFLD with age, sex, metabolic factors, and markers of liver injury, and to identify novel metabolites associated with NAFLD risk, via clinical examination, anthropometric assessment, and targeted metabolomics.

METHODS

Study population

The ethics committee of Wuhan Union Hospital approved the study, and all participants provided written informed consent prior to enrolment. The Wuhan Study is a population-based observational cross-sectional study of participants recruited from the Wuhan Municipality, central China. Participants were recruited from routine health checkups to assess the utility of health checkups in determining the prevalence of and risk factors for NAFLD. Each participant completed a comprehensive

For peer review only

interview and clinical examination that involved the collection of fasting blood and urine samples, abdominal ultrasonography, and anthropometric assessment.

Interview

The interview, which preceded the clinical examination, was conducted by a physician and was designed to obtain information concerning demographic characteristics, medical history, and comorbid conditions. Participants aged over 18 and below 80 years were included if they were not currently being treated for cancer or infectious disease or had undergone surgery in the previous year, and if they had no previous history of cancer or an infectious disease.

Biochemistry

Fasting blood and urine samples were collected on the morning of the clinical examination. Blood triglycerides, glucose, alanine aminotransferase (ALT), aspartate aminotransferase (AST), gamma-glutamyl transferase, alkaline phosphatase, total bilirubin, and uric acid levels were measured using automatic enzymatic procedures (Nanjing Jiancheng Sci-tech, China). Hepatitis B surface antigen (HBsAg), triiodothyronine, thyroxine (T4), and thyroid-stimulating hormone were measured via automatic immunoassays (Roche Diagnostics GmbH, Mannheim, DE).

Diagnosis of NAFLD

Abdominal ultrasonography was performed on all participants by accredited technicians using a Hitachi HI VISION 900 ultrasound machine. Images were stored digitally and evaluated by a senior pathologist. NAFLD was diagnosed by the technician according to ultrasonography [23-25]. Individuals with any of the

For peer review only - http://bmjopen.bmj.com/site/about/guidelines.xhtml

following possible secondary causes of fatty liver were excluded from the analyses: (1) excessive alcohol consumption[26], (2) positive HBsAg or anti-HCV, and (3) use of pharmacological agents historically associated with fatty liver (i.e., amiodarone, corticosteroids, methotrexate, and tamoxifen).[13]

Metabolic covariates

Anthropometric measurements were performed by trained nurses. Body mass index (BMI) was calculated as measured weight (kg) divided by height squared (m^2). Body fat ratio (BFR) was defined as fat weight divided by body weight. The average of two blood pressure measurements, obtained at a single visit in the sitting position after a 10-min rest, was used for analysis.

Metabolic traits were defined: obesity (BMI ≥ 28 kg/m^2), hypertension (blood pressure ≥ 140/90 mmHg or antihypertensive drug treatment), BFR ≥ 25 for women or ≥ 20 for men, blood triglycerides ≥ 1.7 mmol/L, blood fasting glucose ≥ 6.1 mmol/L, liver enzyme elevation (AST ≥ 40 U/L or ALT ≥ 40 U/L), uric acid ≥ 357 μmol/L for women or ≥ 416 μmol/L for men, or impaired OAHOA(< 5 nmol/L).

Targeted LC/MS analysis of SLs and FAHFAs

To identify SLs[21], silica column-affectionate lipid fractions were analyzed by reverse-phase HPLC-MS/MS (Agilent C18 column connected with Thermo Scientific LTQXL; gradients of water to methanol, 10:90 to 0:100) to identify spots present in both control and LD. Identified lipid species of interest were purified by reverse-phase HPLC (Varian Prostar/Agilent C18 column) and subjected to structural analysis.

For peer review only

FAHFAs were measured by HPLC-MS [22] (an Agilent 6410 Triple Quad LC/MS via MRM in negative ionization mode). Briefly, extracted and fractionated samples were reconstituted in 25 ml MeOH; 10 ml was injected for analysis. A Luna C18(2) (Phenomenex) column was used with an in-line filter (Phenomenex). Distinct FAHFAs were resolved via isocratic flow (0.2 ml/min for 120 min, solvent: 93:7 MeOH:H_2O with 5 mM ammonium acetate and 0.01% ammonium hydroxide). Transitions for endogenous OAHOAs were m/z 561.5 → m/z 281.2 (Collision Energy [CE] = 30 V), and m/z 561.5 → m/z 279.2 (CE = 25 V).

STATISTICAL ANALYSES

Baseline analyses were performed using descriptive statistics. U-tests, chi-square tests, or Wilcoxon rank-sum tests (for medians) and Student's t-tests (for means) were used to assess the significance of differences in the distribution of categorical data and continuous data, respectively. To examine associations between traits and NAFLD, we performed binary or multiple logistic regression analyses. We calculated the area under the receiver operating curve (AUC) to assess prediction ability of metabolic markers. A *p*-value of < 0.05 was considered statistically significant unless stated otherwise. Statistical analyses were performed using SPSS software version 17.0 (SPSS Inc., Chicago, IL, USA).

RESULTS

Study population

In total, 1500 participants were enrolled. Twenty-one participants were excluded due to the absence of clinical data. Thus, a total of 1479 study participants were

For peer review only

included in the final analysis, of which 447 reported their age and had no more than one disease, if any (denoted as the "subset"). Patients' general characteristics are shown in Table 1. Men accounted for 40% of the study population (38% of the subset). Participants' mean age was 44.3 ± 11.9 (range 20–74) years.

Prevalence of NAFLD

The prevalence of NAFLD, as determined by ultrasound and biopsy, was 24.7%, (22.8% in the subset).

The peak prevalence of NAFLD in this study was 26.4% and 26.3% between the ages of 30–40 years and 50–60 years, respectively (Table 2).

The prevalence of NAFLD demonstrated an increasing trend with advancing age (OR 1.049, p = 0.607), and after adjustment for sex and metabolic features, NAFLD tended to be inversely related to age (OR 0.844; 95% CI 0.667–1.068; p = 0.157). NAFLD was diagnosed in 37.4% of men and 13.8% of women (p = 0.084). In multivariate analysis, after adjustment for metabolic features and age, male sex was associated with NAFLD (OR 0.287; 95% CI 0.167–0.493; $p < 0.001$, Table 3).

Association between NAFLD and metabolic features

Among metabolic covariates, obesity and hypertension occurred more frequently in participants with advancing age (OR 1.471 and 1.822, respectively; $p < 0.001$). Increased body fat was more prevalent in women than in men (OR 2.042; p < 0.001; [OR 1.754; p = 0.007 in the subset]), and obesity (OR 0.537, $p < 0.001$), increased blood lipids (OR 0.829, p < 0.101), and hypertension (OR 0.769; p < 0.041) were more prevalent in men than in women (OR 0.472, p <0.001; OR 0.902, p < 0.673; and

For peer review only

OR 0.602; p < 0.080, respectively, in the subset).

In univariate analysis, all metabolic and anthropometric traits were significantly associated with NAFLD. In logistic regression analysis, after adjustment for sex, BMI ≥ 24 kg/m^2 (OR 8.494; 95% CI 5.581–12.928; $p < 0.001$), BFR ≥ 25 for women and ≥ 20 for men (OR 1.833; 95% CI 1.286–2.756; $p = 0.001$), triglycerides ≥ 1.7 mmol/L (OR 1.340; 95% CI 1.006–1.785; $p = 0.046$), fasting glucose ≥ 6.1 mmol/L (OR 3.324; 95% CI 1.888–5.850; $p < 0.001$), blood pressure (BP)≥ 140/90 mmHg or antihypertensive drug treatment (OR 1.451, 95% CI 1.069–1.970; $p = 0.017$), uric acid ≥ 357 μmol/L for women and ≥ 416 μmol/L for men (OR 1.448, 95% CI 0.869–2.412; $p = 0.156$), and total OAHOA < 5 nmol/L (OR 1.340, 95% CI 1.006-1.785; p = 0.046) [after adjustment for age stages and sex in the subset, OR for BMI, BFR, and BP: 11.738 (95% CI 5.193–26.530; $p < 0.001$), 2.285 (95% CI 1.067–4.893; p < 0.033) and 1.865 (95% CI 0.910–3.824; p < 0.089), respectively] were independent predictors of NAFLD (Table 3 and Table 4). The prevalence of NAFLD demonstrated an increasing trend with lowering sphingosines (OR 1.448, p = 0.156; Table 4). Substitution of age ranges for continuous data (i.e., absolute age) in logistic regression did not modify the relationships. When each metabolic trait was analyzed independently, after adjustment for age and sex, ORs increased for obesity and hypertension with increasing age, and ORs increased for BFR with increasing age for patients aged <50 years and decreased for patients aged ≥50 years. However, significant interactions were only observed between age and obesity ($p < 0.001$), and between age and hypertension ($p < 0.001$).

Association between NAFLD and liver enzymes

For peer review only

Participants with NAFLD had higher liver enzyme levels than participants without this condition ($p < 0.001$). Abnormal liver enzymes were also significantly associated with NAFLD, independent of sex (OR 3.150, $p < 0.001$). Normal liver enzyme levels, defined according to local guidelines (ALT <40 U/L), were found in 85% of participants with NAFLD.

DISCUSSION

Our results demonstrated that NAFLD was strong associated with metabolic traits including higher body fat, obesity, hyperlipidemia, and impaired fasting glucose. We observed a higher prevalence of NAFLD in men than in women. We conformed the association of impaired uric acid, and abnormal liver enzymes with NAFLD; and further identified impaired total OAHOA as a novel biomarker of NAFLD prevalence.

The average and peak prevalence of NAFLD in this study was 24.7 and more than 26%, respectively, close to the global prevalence of NAFLD, which was 25.24% with highest prevalence in the Middle East(31.79%) and South America(30.45%), and lowest in Africa(13.48%)[27]. The average and peak prevalence of NAFLD in Shanghai [5] was 20.82% and 28.44%, respectively, and those in Guangdong [6] was 17.2% and 27.4%, respectively. The former study was conducted from 2002 to 2003, and the latter in 2005. Our study, performed in 2010, revealed that the average prevalence is increasing, and the peak prevalence occurred at a younger age (30-40 years) than was reported by the previous studies in Shanghai (age of 60-69) [5], Guangdong (age of 60-69) [6] and worldwide (mean age of 70-79)[27]. Thus, more

For peer review only

attention should be given to the health of people in their 30s, when many marry and adopt lifestyle changes, especially in urban China. Unfortunately, we did not have access to information on participants' activity levels, and thus, the effects of physical activity on NAFLD were not assessed. The reasons underlying the observed earlier peak prevalence require further investigation.

We observed that male sex was a risk factor for NAFLD. This finding differs from the results of the Guangdong study [6], and the Rotterdam study [13]. However, this finding is similar to the results of the Shanghai study.

There are several possibilities for this discrepancy. First, the observed sex difference in NAFLD prevalence may be the result of body fat, which is a risk factor for NAFLD and was higher in men than in women in this study. Therefore, male sex may promote NAFLD through mediators such as increased body fat and hormonal change. Second, the sex difference in NAFLD may also result from a lower prevalence of elevated serum triglycerides, glucose, and blood pressure in females compared with males. A lower prevalence of elevated serum triglycerides, glucose and blood pressure in females was reported in a previous study [28]. However, a causal relationship between NAFLD and these serum metabolic markers, as suggested by previous research [29,30], could not be established by our study, given its cross-sectional design. Third, abnormal liver enzymes were more prevalent in men than in women, and both were independent predictors of NAFLD.

We observed that the associations of the identified metabolites with NAFLD risk were pronounced in the Wuhan cohort. These metabolites might play an important role in the development of onset NAFLD. Uric acid was specifically associated with NAFLD in men with type 2 diabetes, independent of insulin resistance and other

For peer review only

metabolic factors [31]. High concentrations of uric acid induce the accumulation of reactive oxygen species in hepatocyte mitochondria, ultimately leading to mitochondrial damage [32], and uric acid was positively correlated with other metabolic traits and was a risk factor for type 2 diabetes mellitus with NAFLD [33].

Serum uric acid (SUA) is the end-product of purine nucleotide catabolism.[34] The SUA subjects have an approximately 2-fold higher risk of NAFLD as defined by ultrasonography. The risk may be independent of age, gender, and obesity (as estimated by BMI and waist circumference). SUA is an independent risk factor in NAFLD in both Uyghurs and Hans in northwestern China.[35] Among subjects with increased SUA levels, women likely showed a greater risk of NAFLD than men.[36]

OAHOAs are present in humans, which are an endogenous branched fatty acid esters of hydroxy fatty acids (FAHFAs) and levels are reduced with obesity and insulin resistance. OAHOA levels in serum correlate highly with whole-body insulin sensitivity. OAHOAs are endogenous GPR120 ligands and may also exert anti-inflammatory effects in vivo through lipid-activated GPCRs, such as GPR120. Atypical integral membrane hydrolases AIG1 and ADTRP degrade bioactive FAHFAs,[37] and branched FAHFAs are preferred substrates of the protein carboxyl ester lipase MODY8.[38] Changes in the levels of these anti-inflammatory metabolites and in their signaling pathways may provide new targets for metabolic and inflammatory diseases [22]. GLUT4 expression and levels of FAHFAs with antidiabetic and anti-inflammatory effects regulates de novo lipogenesis in adipocytes.[39] Compared to healthy controls, FAHFAs significantly decreased in sera of breast cancer patients.[40] Branched FAHFAs protect against colitis by regulating gut innate and adaptive immune responses.[41]

For peer review only

Our study also has some potential limitations. First, we cannot rule out the possibility of biased selection, since non-responders may have had different morbidities. Volunteers in research studies tend to be better educated, healthier, and have better lifestyles [42]. Consequently, estimations of the prevalence of NAFLD may have been biased. Second, only 454 of the 1500 participants provided information on their ages, and we did not obtain data on participants' educational levels or incomes. Third, specific OAHOA isomers [22] require further study.

CONCLUSIONS

NAFLD is common in the study population. In this observational cross-sectional primary survey, we found that NAFLD was associated with male sex, and the peak prevalence occurred at a younger age. In particular, NAFLD was correlated with uric acid. Moreover, we identified OAHOA as a novel biomarker of NAFLD. Further studies are needed to explore the potential factors contributing to these relationships. However, this study is valuable in that it provides a reference on the prevention of NAFLD and related metabolic diseases with the rapid urbanization, technological advancement, and population aging in China.

Contributors Designed the study: Xiu-Ju Zhao. Analyzed data: Xiao-Yu Hu, Yun Li, Long-Quan Li, Yuan Zheng, Jia-Hong Lv, Shu-Chun Huang, Xiu-Ju Zhao. Wrote

For peer review only

and critically review the manuscript: Xiao-Yu Hu, Weinong Zhang, Liang Liu, Ling Zhao, Zhuiguo Liu, Xiu-Ju Zhao.

Conflict of interest: There is no conflict of interest.

Ethics approval The ethics committee of Wuhan Union Hospital approved the study, and all participants provided written informed consent prior to enrolment.

Data sharing statement Data are available on request.

FUNDING STATEMENT: This work was supported in part by the Opening Project of Hubei Key Laboratory of Lipid Chemistry and Nutrition of Oil (201506), and National Nature Science Foundation of China (21602166). They had no role in this study.

For peer review only

REFERENCES

1. Yusuf S, Reddy S, Ounpuu S, *et al*. Global burden of cardiovascular diseases: part I: general considerations, the epidemiologic transition, risk factors, and impact of urbanization. *Circulation* 2001;**104**: 2746-53.
2. Dunn W, Xu R, Wingard D, *et al*. Suspected Nonalcoholic Fatty Liver Disease and Mortality Risk in a Population-Based Cohort Study. *Am J Gastroenterol* 2008;**103**: 2263-71.
3. Ong JP, Pitts A, Younossi ZM. Increased overall mortality and liver-related mortality in non-alcoholic fatty liver disease. *J Hepatol* 2008;**49**: 608-12.
4. Targher G., Day CP, Bonora E. Risk of cardiovascular disease in patients with nonalcoholic fatty liver disease. *N Engl J Med* 2010;**36**: 1341-50.
5. Fan JG, Zhu J, Li X J, *et al*. Prevalence of and risk factors for fatty liver in a general population of Shanghai, China. *J Hepatol* 2005;**43**: 508-14.
6. Zhou YJ, Li YY, Nie YQ, *et al*. Prevalence of fatty liver disease and its risk factors in the population of South China. *World J Gastroenterol* 2007;**13**: 6419-24.
7. Fan JG., Farrell GC. Epidemiology of non-alcoholic fatty liver disease in China. *J Hepatol* 2009;**50**: 204-10.
8. Schwimmer JB, Deutsch R, Kahen T, *et al*. Prevalence of fatty liver in children and adolescents. *Pediatrics* 2006;**118**: 1388-93.
9. Ayonrinde OT, Olynyk JK, Beilin LJ, *et al*. Gender-specific differences in adipose distribution and adipocytokines influence adolescent nonalcoholic fatty liver disease. *Hepatology* 2011;**53**: 800-9.
10. Roy AK, Chatterjee B. Sexual Dimorphism in the Liver. *Annu Rev Physiol* 1983;**45**: 37-50.
11. Sepe A, Tchkonia T, Thomou T, *et al*. Aging and regional differences in fat cell progenitors - a mini-review. *Gerontology* 2011;**57**: 66-75.
12. Takasugi M, Hayakawa K, Arai D, *et al*. Age- and sex-dependent DNA hypomethylation controlled by growth hormone in mouse liver. *Mech Ageing Dev*2013;**134**: 331-7.
13. Koehler EM, Schouten JN, Hansen BE, *et al*. Prevalence and risk factors of non-alcoholic fatty liver disease in the elderly: results from the Rotterdam study. *J Hepatol*2012;**57**: 1305-11.
14. Marchesini G, Brizi M, Morselli-Labate A M, *et al*. Association of nonalcoholic fatty liver disease with insulin resistance. *Am J Med* 1999;**107**: 450-5.
15. Pagano G, Pacini G, Musso G, *et al*. Nonalcoholic steatohepatitis, insulin resistance, and metabolic syndrome: further evidence for an etiologic association. *Hepatology*2002;**35**: 367-72.
16. Cui J, Chen CH, Lo MT, *et al*. Shared genetic effects between hepatic steatosis and fibrosis: A prospective twin study. *Hepatology* 2016; **64**: 1547–58.
17. Bano A, Chaker L, Plompen EPC, *et al*. Thyroid function and the risk of non-alcoholic fatty liver disease: The Rotterdam Study. *J Clin Endocrinol Metab* 2016; **101**: 3204–11.
18. Ting WH, Chien MN, Lo FS, *et al*. Association of cytotoxic T-lymphocyte-associated protein 4 (CTLA4) gene polymorphisms with autoimmune thyroid disease in children and adults: case-control study. *PLoS One* 2016; **11**:e0154394.
19. Menni C, Kastenmüller G, Petersen AK, *et al*. Metabolomic markers reveal novel pathways of ageing and early development in human populations. *Int J Epidemiol* 2013; **42**: 1111–9.

For peer review only

20. Qiu G, Zheng Y, Wang H, et al. Plasma metabolomics identified novel metabolites associated with risk of type 2 diabetes in two prospective cohorts of Chinese adults. *Int J Epidemiol* 2016; **45**: 1507-16.

21. An D, Oh SF, Olszak T, *et al*. Sphingolipids from a symbiotic microbe regulate homeostasis of host intestinal natural killer T cells. *Cell* 2014;**156**: 123-33.

22. Yore MM, Syed I, Moraes-Vieira PM, *et al*. Discovery of a class of endogenous mammalian lipids with anti-diabetic and anti-inflammatory effects. *Cell* 2014;**159**: 318-32.

23. Becker K, Frieling T, Saleh A, *et al*. Resolution of hydatid liver cyst by spontaneous rupture into the biliary tract. *J Hepatol* 1997;**26**: 1408-12.

24. Gustafson DL, Coulson AL, Feng L, *et al*. Use of a medium-term liver focus bioassay to assess the hepatocarcinogenicity of 1,2,4,5-tetrachlorobenzene and 1,4-dichlorobenzene. *Cancer Lett* 1998; **129**: 39-44.

25. Hamaguchi M, Kojima T, Itoh Y, *et al*. The severity of ultrasonographic findings in nonalcoholic fatty liver disease reflects the metabolic syndrome and visceral fat accumulation. *Am J Gastroenterol* 2007; **102**: 2708-15.

26. Baba M, Furuya K, Bandou H, et al. Discrimination of individuals in a general population at high-risk for alcoholic and non-alcoholic fatty liver disease based on liver stiffness: a cross section study. *BMC Gastroenterol* 2011;**11**:70

27. Younossi ZM, Koenig AB, Abdelatif D, et al. Global epidemiology of nonalcoholic fatty liver disease—Meta‐analytic assessment of prevalence, incidence, and outcomes. *Hepatology* 2016;**64**:73-84

28 Bansal SK, Saxena V, Kandpal SuD, *et al*. The prevalence of hypertension and hypertension risk factors in a rural Indian community: A prospective door-to-door study. *J Cardiovasc Dis Res* 2012;**3**: 117-23.

29. Boussageon R, Supper I, Bejan-Angoulvant T, *et al*. Reappraisal of metformin efficacy in the treatment of type 2 diabetes: a meta-analysis of randomised controlled trials. *PLoS Med* 2012; **9**:e1001204.

30. Nordestgaard BG, Palmer TM, Benn M, *et al*. The effect of elevated body mass index on ischemic heart disease risk: causal estimates from a mendelian randomisation approach. *PLoS Med* 2012; **9**:e1001212.

31. Fan N, Zhang L, Xia Z, *et al*. Sex-specific association between serum uric acid and nonalcoholic fatty liver disease in type 2 diabetic patients. *J Diabetes Res* 2016; **2016**: 3805372.

32. Yang Y, Zhou Y, Cheng S, *et al*. Effect of uric acid on mitochondrial function and oxidative stress in hepatocytes. *Genet Mol Res* 2016; **15**: 15028644.

33. Li YL, Xie H, Musha H, *et al*. The risk factor analysis for type 2 diabetes mellitus patients with nonalcoholic fatty liver disease and positive correlation with serum uric acid. *Cell Biochem Biophys* 2015; **72**: 643-7.

34. Liu Z, Que S, Zhou L, *et al*. Dose-response relationship of serum uric acid with metabolic syndrome and non-alcoholic fatty liver disease incidence: a meta-analysis of prospective studies. *Sci Rep* 2015; **5**: 14325

35 Cai W, Wu X, Zhang B, et al. Serum uric acid levels and non-alcoholic fatty liver disease in Uyghur and Han ethnic groups in northwestern China. *Arq Bras Endocrinol Metabol* 2013;**57**:617-22

For peer review only

36. Zhou Y, Wei F, Fan Y. High serum uric acid and risk of nonalcoholic fatty liver disease: A systematic review and meta-analysis. *Clin Biochem* 2016; **49**: 636 – 42.

37 Parsons WH, Kolar MJ, Kamat SS, et al. AIG1 and ADTRP are atypical integral membrane hydrolases that degrade bioactive FAHFAs. *Nat Chem Biol* 2016;**12**:367-72

38. Kolar MJ, Kamat SS, Parsons WH, et al. Branched Fatty Acid Esters of Hydroxy Fatty Acids Are Preferred Substrates of the MODY8 Protein Carboxyl Ester Lipase. *Biochemistry* 2016;**55**:4636-41

39. Moraes-Vieira PM, Saghatelian A, Kahn BB. GLUT4 Expression in Adipocytes Regulates De Novo Lipogenesis and Levels of a Novel Class of Lipids With Antidiabetic and Anti-inflammatory Effects. *DIABETES* 2016;**65**:1808-15

40. Zhu Q-F, Yan J-W, Gao Y, et al. Highly sensitive determination of fatty acid esters of hydroxyl fatty acids by liquid chromatography-mass spectrometry. *J Chromatogr B* 2017;**1061-1062**:34-40

41. Lee J, Moraes-Vieira PM, Castoldi A, et al. Branched Fatty Acid Esters of Hydroxy Fatty Acids (FAHFAs) Protect against Colitis by Regulating Gut Innate and Adaptive Immune Responses. *J Biol Chem* 2016;**291**:22207-17.

42. Sitthi-amorn C, Poshyachinda V. Bias. *Lancet*. 1993;**342**: 286-288

For peer review only

Table 1 Participant characteristics.

a. Subset

Indicators	Total	NAFLD	No NAFLD	*P* value
**n**	447	102 (22.8%)	345 (77.2%)	
**Age (years)**	44.3 ±11.9	44.6 ±10.8	44.0 ±12.1	0.609
**Age stage**	4.0±1.2	4.0±1.2	3.9±1.2	0.608
**Obesity**	235 (51.8%)	94	141	<0.001
**Body fat**	309 (68.1%)	89	220	<0.001
**Blood lipids**	87 (19.2%)	21	66	0.745
**Hypertension**	55 (12.1%)	23	32	0.003
**Male**	171 (38.3%)	64	107	<0.001
**Elevated liver enzymes**	18 (4.0%)	7	11	0.174

Age stages of 20-30, 30-40, 40-50, 50-60, ≥60 are 2, 3, 4, 5, 6.

b Total study population

Indicators	Total	NAFLD	No NAFLD	*P* value
**n**	1479	365 (24.7%)	1114 (75.3%)	
**Obesity**	809 (54.7%)	334	475	<0.001
**Body fat**	1003 (67.8%)	309	694	<0.001

For peer review only

**Blood lipid**	458 (30.9%)	163	295	<0.001
**High blood glucose**	71 (4.8%)	43	28	<0.001
**Hypertension**	314 (21.2%)	129	185	<0.001
**Abnormal uric acid**	83 (5.6%)	38	45	<0.001
**Male**	590 (39.8%)	214	376	<0.001
**Impaired liver function**	32 (0.2%)	12	20	0.142
**Elevated liver enzymes**	110 (0.7%)	53	57	<0.001

Conditions were defined as follows: obesity (BMI ≥ 24 kg/m^2), hypertension (blood pressure ≥ 140/90 mmHg or antihypertensive drug treatment), Body fat ratio ≥ 25 for women or ≥ 20 for men, blood triglycerides ≥ 1.7 mmol/L, blood fasting glucose ≥ 5.6 mmol/L, impaired liver function (positive HBsAg), liver enzyme elevation (AST ≥ 40 U/L or ALT ≥ 40 U/L), uric acid ≥ 357 μmol/L for women or ≥ 416 μmol/L for men.

Mean values are provided with standard deviation, unless otherwise noted as n (%). Differences between participants with and without NAFLD were evaluated with t-tests or the Wilcoxon-Mann-Whitney test for continuous variables and the chi-squared test for categorical variables. NAFLD, non-alcoholic fatty liver disease.

For peer review only

Table 2. NAFLD prevalence across age range.

Age range (years)	Participants, n	Participants with NAFLD, n	NAFLD prevalence,%	U-test
20-30	64	10	15.6	1.15
30-40	102	27	26.4	0.85
40-50	121	26	21.5	0.13
50-60	114	30	26.3	1.00
≥60	53	9	17.0	0.78
Total	454	102	22.4	1.64 (α=0.1)

NAFLD, non-alcoholic fatty liver disease.

For peer review only

Table 3. Multivariate adjusted models for liver disease subtypes.

a Subset

Variables	OR	*p* value
Sex (female)	0.267	<0.001
Obesity	16.667	<0.001
Body fat	3.859	<0.001
Elevated liver enzyme	2.237	0.105
Hypertension	2.848	0.001

b The whole

Variables	OR	*p* value
Sex (female)	0.359	<0.001
Obesity	14.109	<0.001
Body fat	3.292	<0.001
Blood lipid	2.240	<0.001
Blood glucose	5.185	<0.001
Impaired liver function	1.859	0.094
Elevated liver enzyme	3.150	<0.001
Hypertension	2.739	<0.001

Conditions were defined as follows: female sex=1, obesity (BMI ≥ 24 kg/m^2), hypertension (blood pressure ≥ 140/90 mmHg or antihypertensive drug treatment), body fat ratio ≥ 25 for women or ≥ 20 for men, blood fasting

For peer review only

glucose ≥ 6.1 mmol/L, liver enzyme elevation (AST ≥ 40 U/L or ALT ≥ 40 U/L)

NAFLD, non-alcoholic fatty liver disease.

Odds ratios and the corresponding p values derived from binary or multiple logistic regression analyses using SPSS software version 17.0 (SPSS Inc., Chicago, IL, USA)

For peer review only

Table 4. Metabolites associated with non-alcoholic fatty liver disease.

**Metabolite**	**OR, *P* value**	**AUC, *P* value**
**Uric acid**	2.755 <0.001	0.579 <0.001
**Sphingosine**	1.448 0.156	0.489 0.760
**OAHOA**	1.340 0.046	0.612 0.001

Conditions were defined: Sphingosine < 2 nmol/L, OAHOA (oleic acid -hydroxy oleic acid) < 5 nmol/L.

NAFLD, non-alcoholic fatty liver disease.

Odds ratios (OR), area under curve (AUC) and the corresponding p values derived from binary or multiple logistic regression, or ROC analyses using SPSS software version 17.0 (SPSS Inc., Chicago, IL, USA)

For peer review only

CONSORT 2010 checklist of information to include when reporting a randomised trial*

Section/Topic	Item No	Checklist item	Reported on page No
**Title and abstract**			
	1a	Identification as a randomised trial in the title	1
	1b	Structured summary of trial design, methods, results, and conclusions (for specific guidance see CONSORT for abstracts)	3
**Introduction**			
Background and objectives	2a	Scientific background and explanation of rationale	5-6
	2b	Specific objectives or hypotheses	5-6
**Methods**			
Trial design	3a	Description of trial design (such as parallel, factorial) including allocation ratio	6
	3b	Important changes to methods after trial commencement (such as eligibility criteria), with reasons	6
Participants	4a	Eligibility criteria for participants	6
	4b	Settings and locations where the data were collected	6
Interventions	5	The interventions for each group with sufficient details to allow replication, including how and when they were actually administered	7
Outcomes	6a	Completely defined pre-specified primary and secondary outcome measures, including how and when they were assessed	7
	6b	Any changes to trial outcomes after the trial commenced, with reasons	7
Sample size	7a	How sample size was determined	6
	7b	When applicable, explanation of any interim analyses and stopping guidelines	6
Randomisation:			
Sequence generation	8a	Method used to generate the random allocation sequence	7
	8b	Type of randomisation; details of any restriction (such as blocking and block size)	7
Allocation concealment mechanism	9	Mechanism used to implement the random allocation sequence (such as sequentially numbered containers), describing any steps taken to conceal the sequence until interventions were assigned	7
Implementation	10	Who generated the random allocation sequence, who enrolled participants, and who assigned participants to interventions	7
Blinding	11a	If done, who was blinded after assignment to interventions (for example, participants, care providers, those	-

For peer review only - http://bmjopen.bmj.com/site/about/guidelines.xhtml

		assessing outcomes) and how	
	11b	If relevant, description of the similarity of interventions	-
Statistical methods	12a	Statistical methods used to compare groups for primary and secondary outcomes	8
	12b	Methods for additional analyses, such as subgroup analyses and adjusted analyses	8
**Results**			
Participant flow (a diagram is strongly recommended)	13a	For each group, the numbers of participants who were randomly assigned, received intended treatment, and were analysed for the primary outcome	7
	13b	For each group, losses and exclusions after randomisation, together with reasons	7
Recruitment	14a	Dates defining the periods of recruitment and follow-up	7
	14b	Why the trial ended or was stopped	7
Baseline data	15	A table showing baseline demographic and clinical characteristics for each group	7
Numbers analysed	16	For each group, number of participants (denominator) included in each analysis and whether the analysis was by original assigned groups	7
Outcomes and estimation	17a	For each primary and secondary outcome, results for each group, and the estimated effect size and its precision (such as 95% confidence interval)	7
	17b	For binary outcomes, presentation of both absolute and relative effect sizes is recommended	7
Ancillary analyses	18	Results of any other analyses performed, including subgroup analyses and adjusted analyses, distinguishing pre-specified from exploratory	8
Harms	19	All important harms or unintended effects in each group (for specific guidance see CONSORT for harms)	7
**Discussion**			
Limitations	20	Trial limitations, addressing sources of potential bias, imprecision, and, if relevant, multiplicity of analyses	18
Generalisability	21	Generalisability (external validity, applicability) of the trial findings	7
Interpretation	22	Interpretation consistent with results, balancing benefits and harms, and considering other relevant evidence	18
**Other information**			
Registration	23	Registration number and name of trial registry	-
Protocol	24	Where the full trial protocol can be accessed, if available	-
Funding	25	Sources of funding and other support (such as supply of drugs), role of funders	2

*We strongly recommend reading this statement in conjunction with the CONSORT 2010 Explanation and Elaboration for important clarifications on all the items. If relevant, we also recommend reading CONSORT extensions for cluster randomised trials, non-inferiority and equivalence trials, non-pharmacological treatments, herbal interventions, and pragmatic trials. Additional extensions are forthcoming: for those and for up to date references relevant to this checklist, see www.consort-statement.org.

For peer review only - http://bmjopen.bmj.com/site/about/guidelines.xhtml

BMJ Open

Risk Factors and Biomarkers of Non-alcoholic Fatty Liver Disease: an observational cross-sectional population survey

Journal:	*BMJ Open*
Manuscript ID	bmjopen-2017-019974.R2
Article Type:	Research
Date Submitted by the Author:	22-Jan-2018
Complete List of Authors:	Hu, Xiao-Yu ; Wuhan Polytechnic University, School of Biology and Pharmaceutical Engineering<br>Li, Yun ; Wuhan Polytechnic University, School of Biology and Pharmaceutical Engineering<br>Li, Long-Quan ; Wuhan Polytechnic University, , School of Biology and Pharmaceutical Engineering<br>Zheng, Yuan ; Wuhan Polytechnic University, , School of Biology and Pharmaceutical Engineering<br>Lv, Jia-Hong ; Wuhan Polytechnic University, , School of Biology and Pharmaceutical Engineering<br>Huang , Shu-Chun; Wuhan Polytechnic University, School of Biology and Pharmaceutical Engineering<br>Zhang, Wei-Nong ; Wuhan Polytechnic University, School of Biology and Pharmaceutical Engineering<br>Liu, Liang; Wuhan Polytechnic University, School of Biology and Pharmaceutical Engineering<br>Zhao, Ling; Wuhan Polytechnic University, School of Biology and Pharmaceutical Engineering<br>Liu, Zhiguo; Wuhan Polytechnic University, School of Biology and Pharmaceutical Engineering<br>Zhao, Xiu-Ju
**Primary Subject Heading**:	Public health
Secondary Subject Heading:	Gastroenterology and hepatology, Epidemiology
Keywords:	EPIDEMIOLOGY, Hepatology < INTERNAL MEDICINE, PUBLIC HEALTH

SCHOLARONE™
Manuscripts

Risk Factors and Biomarkers of Non-alcoholic Fatty Liver Disease: an observational cross-sectional population survey

Xiao-Yu Hu[1], Yun Li[1], Long-Quan Li[1], Yuan Zheng[1], Jia-Hong Lv[1], Shu-Chun Huang[1], Weinong Zhang[1], Liang Liu[1], Ling Zhao[1], Zhuiguo Liu[1], Xiu-Ju Zhao[1,2,3*]

1 School of Biology and Pharmaceutical Engineering, Wuhan Polytechnic University, 68 South Xuefu Road, Changqing Garden, Wuhan, Hubei 430023, P. R. China

2 Hubei Key Laboratory of Lipid Chemistry and Nutrition of Oil, Oil Crops Research Institute of the Chinese Academy of Agricultural Sciences, Wuhan, Hubei 430062, P. R. China

3 Department of Nutrition and Food Science, Texas A&M University, College Station, TX 77843, U.S.A.

Corresponding

Xiu-Ju Zhao

E-mail: dzrdez@163.com, Tel. and Fax: +862783956793, School of Biology and Pharmaceutical Engineering, Wuhan Polytechnic University, 68 South Xuefu Road, Changqing Garden, Wuhan 430023, P. R. China

Abbreviations: NAFLD, non-alcoholic fatty liver disease; BMI, body mass index; BFR, body fat ratio; HBsAg, hepatitis B surface antigen; ALT, alanine aminotransferase; AST, aspartate aminotransferase

For peer review only

ABSTRACT

Objectives Non-alcoholic fatty liver disease (NAFLD) is a major public health burden in China and its prevalence is increasing. This study aimed to determine risk factors and biomarkers of NAFLD.

Design An observational cross-sectional primary survey.

Setting Central China.

Participants 1479 aged over 18 and below 80 years, not currently being treated for cancer or infectious disease or no surgery in the previous year, and no previous history of cancer or an infectious disease. Participants underwent clinical examination, metabolomic assay, and anthropometric assessment. Univariate and logistic regression analyses were used to assess associations between covariates and NAFLD.

Main outcome measures Risk factors and metabolic biomarkers including sex, body mass index, hypertension, body fat ratio, blood triglycerides, blood fasting glucose, liver enzyme elevation, uric acid, and oleic acid-hydroxy oleic acid.

Results Data from the 447 participants who reported their age reported their age and had no more than one disease, if any, (mean age 44.3 ± 11.9 years) were analyzed. The prevalence of NAFLD was 24.7%. Male (OR 3.484; 95% CI 2.028–5.988), body mass index ≥ 24 kg/m^2 (OR 8.494; 95% CI 5.581–12.928), body fat ratio (≥ 25, women; ≥ 20, men) (OR 1.833; 95% CI 1.286–2.756), triglycerides ≥ 1.7 mmol/L (OR 1.340; 95% CI 1.006–1.785), fasting glucose ≥ 6.1 mmol/L (OR 3.324; 95% CI 1.888–5.850), blood pressure ≥ 140/90 mmHg or antihypertensive drug treatment (OR 1.451, 95% CI 1.069–1.970), uric acid (≥ 357 μmol/L, women; ≥ 416 μmol/L, men) (OR 2.755, 95% CI 2.009–3.778), and oleic acid-hydroxy oleic acid (OAHOA < 5 nmol/L) (OR 1.340, 95% CI 1.006-1.785) were independent predictors of NAFLD (all $p < 0.05$). These results were verified by the whole 1479 participants.

For peer review only

Conclusions NAFLD was common among the study participants. In particular, NAFLD was correlated with uric acid. We identified OAHOA as a novel marker of NAFLD prevalence. It provides a reference on the prevention of NAFLD and related metabolic diseases with the rapid urbanization, technological advancement, and population aging in China over the recent decades.

Strengths and limitations of this study

Risk factors of NAFLD in central China.

Biomarkers of NAFLD using metabolomics.

Prediction ability of metabolic markers using ROC.

No information on education.

Keywords: uric acid; oleic acid-hydroxy oleic acid; male; non-alcoholic fatty liver disease (NAFLD)

For peer review only

INTRODUCTION

China has the world's largest population and is undergoing rapid economic growth and social reform. This advancement has paralleled demographic, lifestyle, and cultural changes that have exerted notable effects on the health profile of China's residents and placed significant constraints on the country's healthcare system. [1]

Such changes are apparent in major cities of central China, such as Wuhan. In China, the prevalence of non-alcoholic fatty liver disease (NAFLD) is increasing and it is currently the most common chronic disease among Chinese adults, with rates exceeding those of equally important epidemics including obesity, hypertension, and type II diabetes. NAFLD has been associated with an increase in overall and cardiovascular morbidity and mortality.[2-4] It has been estimated that NAFLD will be the most common indicator for liver transplantation and regeneration in the coming decades.[5-7] In addition, the prevalence and impact of NAFLD in China is expected to increase as a result of population aging and the continual increase in obesity and hypertension rates with reforming and open-up over the recent decades. Therefore, NAFLD is a major public health burden.

The reported prevalence of NAFLD among Chinese adults ranges from 15% to 30%.[5-7] NAFLD prevalence increases with age, most notably from the fourth decade of life onward (40 - 60 years of age).[5,6,8,9] The prevalence and risk factors for NAFLD may vary across different ages and between male and female populations as a result of metabolic changes including fat redistribution and endocrine function.[10-13] However, the association of age and sex with NAFLD in central China is still unclear. Furthermore, the association of hypertension, obesity,

For peer review only

hyperlipidemia, insulin resistance, and diabetes with NAFLD has been widely investigated in adult cohorts [14-16], and these metabolic traits are now well-accepted risk factors for NAFLD. However, limited data are available concerning the association of NAFLD with other covariant traits [17,18]. Metabolomics provides the integrated information on biological status and can investigate the molecular variations with disease phenotyping[19,20]; several markers for NAFLD have been identified previously[5,6]. Sphingolipids (SLs) and branched fatty acid esters of hydroxy fatty acids (FAHFAs) have reported to protect against autoimmune and allergic disorders and type 2 diabetes, respectively[21,22]; the effects of SLs and FAHFAs on NAFLD are largely unclear.

The aim of the present study was to investigate the prevalence of NAFLD, to elucidate the association of NAFLD with age, sex, metabolic factors, and markers of liver injury, and to identify novel metabolites associated with NAFLD risk, via clinical examination, anthropometric assessment, and targeted metabolomics.

METHODS

Study population

The ethics committee of Wuhan Union Hospital approved the study, and all participants provided written informed consent prior to enrolment. The Wuhan Study is an observational cross-sectional study of participants recruited from the Wuhan Municipality, central China. Participants were recruited from routine health checkups to assess the utility of health checkups in determining the prevalence of and risk factors for NAFLD. Each participant completed a comprehensive interview and

For peer review only

clinical examination that involved the collection of fasting blood and urine samples, abdominal ultrasonography, and anthropometric assessment.

Interview

The interview, which preceded the clinical examination, was conducted by a physician and was designed to obtain information concerning demographic characteristics, medical history, and comorbid conditions. Participants aged over 18 and below 80 years were included if they were not currently being treated for cancer or infectious disease or had undergone surgery in the previous year, and if they had no previous history of cancer or an infectious disease.

Biochemistry

Fasting blood and urine samples were collected on the morning of the clinical examination. Blood triglycerides, glucose, alanine aminotransferase (ALT), aspartate aminotransferase (AST), gamma-glutamyl transferase, alkaline phosphatase, total bilirubin, and uric acid levels were measured using automatic enzymatic procedures (Nanjing Jiancheng Sci-tech, China). Hepatitis B surface antigen (HBsAg), triiodothyronine, thyroxine (T4), and thyroid-stimulating hormone were measured via automatic immunoassays (Roche Diagnostics GmbH, Mannheim, DE).

Diagnosis of NAFLD

Abdominal ultrasonography was performed on all participants by accredited technicians using a Hitachi HI VISION 900 ultrasound machine. Images were stored digitally and evaluated by a senior pathologist. NAFLD was diagnosed by the technician according to ultrasonography [23-25]. Individuals with any of the

For peer review only

following possible secondary causes of fatty liver were excluded from the analyses: (1) excessive alcohol consumption[26], (2) positive HBsAg or anti-HCV, and (3) use of pharmacological agents historically associated with fatty liver (i.e., amiodarone, corticosteroids, methotrexate, and tamoxifen).[13]

Metabolic covariates

Anthropometric measurements were performed by trained nurses. Body mass index (BMI) was calculated as measured weight (kg) divided by height squared (m^2). Body fat ratio (BFR) was defined as fat weight divided by body weight. The average of two blood pressure measurements, obtained at a single visit in the sitting position after a 10-min rest, was used for analysis.

Metabolic traits were defined: obesity (BMI ≥ 28 kg/m^2), hypertension (blood pressure ≥ 140/90 mmHg or antihypertensive drug treatment), BFR ≥ 25 for women or ≥ 20 for men, blood triglycerides ≥ 1.7 mmol/L, blood fasting glucose ≥ 6.1 mmol/L, liver enzyme elevation (AST ≥ 40 U/L or ALT ≥ 40 U/L), uric acid ≥ 357 μmol/L for women or ≥ 416 μmol/L for men, or impaired OAHOA(< 5 nmol/L).

Targeted LC/MS analysis of SLs and FAHFAs

To identify SLs[21], silica column-affectionate lipid fractions were analyzed by reverse-phase HPLC-MS/MS (Agilent C18 column connected with Thermo Scientific LTQXL; gradients of water to methanol, 10:90 to 0:100) to identify spots present in both control and LD. Identified lipid species of interest were purified by reverse-phase HPLC (Varian Prostar/Agilent C18 column) and subjected to structural analysis.

For peer review only

FAHFAs were measured by HPLC-MS [22] (an Agilent 6410 Triple Quad LC/MS via MRM in negative ionization mode). Briefly, Extracted and fractionated samples were reconstituted in 25 ml MeOH; 10 ml was injected for analysis. A Luna C18(2) (Phenomenex) column was used with an in-line filter (Phenomenex). Distinct FAHFAs were resolved via isocratic flow (0.2 ml/min for 120 min, solvent: 93:7 MeOH:H_2O with 5 mM ammonium acetate and 0.01% ammonium hydroxide). Transitions for endogenous OAHOAs were m/z 561.5 → m/z 281.2 (Collision Energy [CE] = 30 V), and m/z 561.5 → m/z 279.2 (CE = 25 V).

STATISTICAL ANALYSES

Baseline analyses were performed using descriptive statistics. U-tests, chi-square tests, or Wilcoxon rank-sum tests (for medians) and Student's t-tests (for means) were used to assess the significance of differences in the distribution of categorical data and continuous data, respectively. To examine associations between traits and NAFLD, we performed binary or multiple logistic regression analyses. We calculated the area under the receiver operating curve (AUC) to assess prediction ability of metabolic markers. A p-value of < 0.05 was considered statistically significant unless stated otherwise. Statistical analyses were performed using SPSS software version 17.0 (SPSS Inc., Chicago, IL, USA).

RESULTS

Study population

In total, 1500 participants were enrolled. Twenty-one participants were excluded due to the absence of clinical data. Thus, a total of 1479 study participants were

For peer review only

included in the final analysis, of which 447 reported their age and had no more than one disease, if any (denoted as the "subset"). Patients' general characteristics are shown in Table 1. Men accounted for 40% of the study population (38% of the subset). Participants' mean age was 44.3 ± 11.9 (range 20–74) years.

Prevalence of NAFLD

The prevalence of NAFLD, as determined by ultrasound and biopsy, was 24.7%, (22.8% in the subset).

The peak prevalence of NAFLD in this study was 26.4% and 26.3% between the ages of 30–40 years and 50–60 years, respectively (Table 2).

The prevalence of NAFLD demonstrated an increasing trend with advancing age (OR 1.049, $p = 0.607$), and after adjustment for sex and metabolic features, NAFLD tended to be inversely related to age (OR 0.844; 95% CI 0.667–1.068; $p = 0.157$). NAFLD was diagnosed in 37.4% of men and 13.8% of women ($p = 0.084$). In multivariate analysis, after adjustment for metabolic features and age, male sex was associated with NAFLD (OR 3.484; 95% CI 2.028–5.988; $p < 0.001$, Table 3a).

Association between NAFLD and metabolic features

Among metabolic covariates, obesity and hypertension occurred more frequently in participants with advancing age (OR 1.471 and 1.822, respectively; $p < 0.001$). Increased body fat was more prevalent in women than in men (OR 2.042; p < 0.001; [OR 1.754; p = 0.007 in the subset]), and obesity (OR 0.537, $p < 0.001$), increased blood lipids (OR 0.829, p < 0.101), and hypertension (OR 0.769; p < 0.041) were more prevalent in men than in women (OR 0.472, p <0.001; OR 0.902, p < 0.673; and

For peer review only

OR 0.602; p < 0.080, respectively, in the subset).

In univariate analysis, all metabolic and anthropometric traits were significantly associated with NAFLD. In logistic regression analysis, after adjustment for sex, BMI ≥ 24 kg/m^2 (OR 8.494; 95% CI 5.581–12.928; p < 0.001), BFR ≥ 25 for women and ≥ 20 for men (OR 1.833; 95% CI 1.286–2.756; p = 0.001), triglycerides ≥ 1.7 mmol/L (OR 1.340; 95% CI 1.006–1.785; p = 0.046), fasting glucose ≥ 6.1 mmol/L (OR 3.324; 95% CI 1.888–5.850; p < 0.001), blood pressure (BP)≥ 140/90 mmHg or antihypertensive drug treatment (OR 1.451, 95% CI 1.069–1.970; p = 0.017), uric acid ≥ 357 μmol/L for women and ≥ 416 μmol/L for men (OR 2.755, 95% CI 2.009–3.778; p < 0.001), and total OAHOA < 5 nmol/L (OR 1.340, 95% CI 1.006-1.785; p = 0.046) [after adjustment for age stages and sex in the subset, ORs for BMI, BFR, and BP: 11.738 (95% CI 5.193–26.530; p < 0.001), 2.285 (95% CI 1.067–4.893; p = 0.033) and 1.865 (95% CI 0.910–3.824; p = 0.089), respectively] were independent predictors of NAFLD (Table 3a, b and Table 4). The prevalence of NAFLD demonstrated an increasing trend with lowering sphingosines (OR 1.448, p = 0.156; Table 4). Substitution of age ranges for continuous data (i.e., absolute age) in logistic regression did not modify the relationships. When each metabolic trait was analyzed independently, after adjustment for age and sex, ORs increased for obesity and hypertension with increasing age, and ORs increased for BFR with increasing age for patients aged <50 years and decreased for patients aged ≥50 years. However, significant interactions were only observed between age and obesity (p < 0.001), and between age and hypertension (p < 0.001).

Association between NAFLD and liver enzymes

For peer review only

Participants with NAFLD had higher liver enzyme levels than participants without this condition ($p < 0.001$). Abnormal liver enzymes were also significantly associated with NAFLD, independent of sex (OR 3.150, $p < 0.001$). Normal liver enzyme levels, defined according to local guidelines (ALT <40 U/L), were found in 85% of participants with NAFLD.

DISCUSSION

For peer review only

Our results demonstrated that NAFLD was strong associated with metabolic traits including higher body fat, obesity, hyperlipidemia, and impaired fasting glucose. We observed a higher prevalence of NAFLD in men than in women. We conformed the association of impaired uric acid, and abnormal liver enzymes with NAFLD; and further identified impaired total OAHOA as a novel biomarker of NAFLD prevalence.

The average and peak prevalence of NAFLD in this study was 24.7 and more than 26%, respectively, close to the global prevalence of NAFLD, which was 25.24% with highest prevalence in the Middle East(31.79%) and South America(30.45%), and lowest in Africa(13.48%)[27]. The average and peak prevalence of NAFLD in Shanghai [5] was 20.82% and 28.44%, respectively, and those in Guangdong [6] was 17.2% and 27.4%, respectively. The former study was conducted from 2002 to 2003, and the latter in 2005. Our study, performed in 2010, revealed that the average prevalence is increasing, and the peak prevalence occurred at a younger age (30-40 years) than was reported by the previous studies in Shanghai (age of 60-69) [5], Guangdong (age of 60-69) [6] and worldwide (mean age of 70-79)[27]. Thus, more

attention should be given to the health of people in their 30s, when many marry and adopt lifestyle changes, especially in urban China. Unfortunately, we did not have access to information on participants' activity levels, and thus, the effects of physical activity on NAFLD were not assessed. The reasons underlying the observed earlier peak prevalence require further investigation.

We observed that male sex was a risk factor for NAFLD. This finding differs from the results of the Guangdong study [6], and the Rotterdam study [13]. However, this finding is similar to the results of the Shanghai study.

There are several possibilities for this discrepancy. First, the observed sex difference in NAFLD prevalence may be the result of body fat, which is a risk factor for NAFLD and was higher in men than in women in this study. Therefore, male sex may promote NAFLD through mediators such as increased body fat and hormonal change. Second, the sex difference in NAFLD may also result from a lower prevalence of elevated serum triglycerides, glucose, and blood pressure in females compared with males. A lower prevalence of elevated serum triglycerides, glucose and blood pressure in females was reported in a previous study [28]. However, a causal relationship between NAFLD and these serum metabolic markers, as suggested by previous research [29,30], could not be established by our study, given its cross-sectional design. Third, abnormal liver enzymes were more prevalent in men than in women, and both were independent predictors of NAFLD.

We observed that the associations of the identified metabolites with NAFLD risk were pronounced in the Wuhan cohort. These metabolites might play an important role in the development of onset NAFLD. Uric acid was specifically associated with NAFLD in men with type 2 diabetes, independent of insulin resistance and other

For peer review only

metabolic factors [31]. High concentrations of uric acid induce the accumulation of reactive oxygen species in hepatocyte mitochondria, ultimately leading to mitochondrial damage [32], and uric acid was positively correlated with other metabolic traits and was a risk factor for type 2 diabetes mellitus with NAFLD [33].

Serum uric acid (SUA) is the end-product of purine nucleotide catabolism.[34] The SUA subjects have an approximately 2-fold higher risk of NAFLD as defined by ultrasonography. The risk may be independent of age, gender, and obesity (as estimated by BMI and waist circumference). SUA is an independent risk factor in NAFLD in both Uyghurs and Hans in northwestern China.[35] Among subjects with increased SUA levels, women likely showed a greater risk of NAFLD than men.[36]

OAHOAs are present in humans, which are an endogenous branched fatty acid esters of hydroxy fatty acids (FAHFAs) and levels are reduced with obesity and insulin resistance. OAHOA levels in serum correlate highly with whole-body insulin sensitivity. OAHOAs are endogenous GPR120 ligands and may also exert anti-inflammatory effects in vivo through lipid-activated GPCRs, such as GPR120. Atypical integral membrane hydrolases AIG1 and ADTRP degrade bioactive FAHFAs,[37] and branched FAHFAs are preferred substrates of the protein carboxyl ester lipase MODY8.[38] Changes in the levels of these anti-inflammatory metabolites and in their signaling pathways may provide new targets for metabolic and inflammatory diseases [22]. GLUT4 expression and levels of FAHFAs with antidiabetic and anti-inflammatory effects regulates de novo lipogenesis in adipocytes.[39] Compared to healthy controls, FAHFAs significantly decreased in sera of breast cancer patients.[40] Branched FAHFAs protect against colitis by regulating gut innate and adaptive immune responses.[41]

For peer review only

Our study also has some potential limitations. First, we cannot rule out the possibility of biased selection, since non-responders may have had different morbidities. Volunteers in research studies tend to be better educated, healthier, and have better lifestyles [42]. Consequently, estimations of the prevalence of NAFLD may have been biased. Second, only 447 of the 1479 participants provided information on their ages, and we did not obtain data on participants' educational levels or incomes. Third, specific OAHOA isomers [22] require further study.

CONCLUSIONS

NAFLD is common in the study population. In this observational cross-sectional primary survey, we found that NAFLD was associated with male sex, and the peak prevalence occurred at a younger age. In particular, NAFLD was correlated with uric acid. Moreover, we identified OAHOA as a novel biomarker of NAFLD. Further studies are needed to explore the potential factors contributing to these relationships. However, this study is valuable in that it provides a reference on the prevention of NAFLD and related metabolic diseases with the rapid urbanization, technological advancement, and population aging in China.

Contributors Designed the study: Xiu-Ju Zhao. Analyzed data: Xiao-Yu Hu, Yun Li, Long-Quan Li, Yuan Zheng, Jia-Hong Lv, Shu-Chun Huang, Xiu-Ju Zhao. Wrote

For peer review only

and critically review the manuscript: Xiao-Yu Hu, Weinong Zhang, Liang Liu, Ling Zhao, Zhuiguo Liu, Xiu-Ju Zhao.

Conflict of interest: There is no conflict of interest.

Ethics approval The ethics committee of Wuhan Union Hospital approved the study, and all participants provided written informed consent prior to enrolment.

Data sharing statement Data are available on request.

FUNDING STATEMENT: This work was supported in part by the Opening Project of Hubei Key Laboratory of Lipid Chemistry and Nutrition of Oil (201506), and National Nature Science Foundation of China (21602166). They had no role in this study.

For peer review only

REFERENCES

1. Yusuf S, Reddy S, Ounpuu S, Anand S. Global burden of cardiovascular diseases: part I: general considerations, the epidemiologic transition, risk factors, and impact of urbanization. *Circulation* 2001;**104**: 2746-53.

2. Dunn W, Xu R, Wingard D, Rogers C, Angulo P, Younossi Z *et al*. Suspected Nonalcoholic Fatty Liver Disease and Mortality Risk in a Population-Based Cohort Study. *Am J Gastroenterol* 2008;**103**: 2263-71.

3. Ong JP, Pitts A, Younossi ZM. Increased overall mortality and liver-related mortality in non-alcoholic fatty liver disease. *J Hepatol* 2008;**49**: 608-12.

4. Targher G., Day CP, Bonora E. Risk of cardiovascular disease in patients with nonalcoholic fatty liver disease. *N Engl J Med* 2010;**36**: 1341-50.

5. Fan JG, Zhu J, Li X J, Chen L, Li L, Dai F *et al*. Prevalence of and risk factors for fatty liver in a general population of Shanghai, China. *J Hepatol* 2005;**43**: 508-14.

6. Zhou YJ, Li YY, Nie YQ, Ma JX, Lu LG, Shi SL *et al*. Prevalence of fatty liver disease and its risk factors in the population of South China. *World J Gastroenterol* 2007;**13**: 6419-24.

7. Fan JG., Farrell GC. Epidemiology of non-alcoholic fatty liver disease in China. *J Hepatol* 2009;**50**: 204-10.

8. Schwimmer JB, Deutsch R, Kahen T, Lavine J E, Stanley C, Behling C. Prevalence of fatty liver in children and adolescents. *Pediatrics* 2006;**118**: 1388-93.

9. Ayonrinde OT, Olynyk JK, Beilin LJ, Mori TA, Pennell CE, de Klerk N *et al*. Gender-specific differences in adipose distribution and adipocytokines influence adolescent nonalcoholic fatty liver disease. *Hepatology* 2011;**53**: 800-9.

10. Roy AK, Chatterjee B. Sexual Dimorphism in the Liver. *Annu Rev Physiol* 1983;**45**: 37-50.

11. Sepe A, Tchkonia T, Thomou T, Zamboni M, Kirkland JL. Aging and regional differences in fat cell progenitors - a mini-review. *Gerontology* 2011;**57**: 66-75.

12. Takasugi M, Hayakawa K, Arai D, Shiota K. Age- and sex-dependent DNA hypomethylation controlled by growth hormone in mouse liver. *Mech Ageing Dev* 2013;**134**: 331-7.

13. Koehler EM, Schouten JN, Hansen BE, van Rooij FJ, Hofman A, Stricker BH, *et al*. Prevalence and risk factors of non-alcoholic fatty liver disease in the elderly: results from the Rotterdam study. *J Hepatol* 2012;**57**: 1305-11.

14. Marchesini G, Brizi M, Morselli-Labate A M, Bianchi G, Bugianesi E, McCullough AJ *et al*. Association of nonalcoholic fatty liver disease with insulin resistance. *Am J Med* 1999;**107**: 450-5.

15. Pagano G, Pacini G, Musso G, Gambino R, Mecca F, Depetris N *et al*. Nonalcoholic steatohepatitis, insulin resistance, and metabolic syndrome: further evidence for an etiologic association. *Hepatology* 2002;**35**: 367-72.

16. Cui J, Chen CH, Lo MT, Schork N, Bettencourt R, Gonzalez MP *et al*. Shared genetic effects between hepatic steatosis and fibrosis: A prospective twin study. *Hepatology* 2016; **64**: 1547–58.

17. Bano A, Chaker L, Plompen EPC, Hofman A, Dehghan A, Franco OH *et al*. Thyroid function and the risk of non-alcoholic fatty liver disease: The Rotterdam Study. *J Clin Endocrinol Metab*. 2016; **101**: 3204–11.

18. Ting WH, Chien MN, Lo FS, Wang CH, Huang CY, Lin CL *et al*. Association of cytotoxic

For peer review only

T-lymphocyte-associated protein 4 (CTLA4) gene polymorphisms with autoimmune thyroid disease in children and adults: case-control study. *PLoS One*. 2016; **11**:e0154394.

19. Menni C, Kastenmüller G, Petersen AK, Bell JT, Psatha M, Tsai PC *et al*. Metabolomic markers reveal novel pathways of ageing and early development in human populations. *Int J Epidemiol* 2013; **42**: 1111–9.

20. Qiu, G. et al. Plasma metabolomics identified novel metabolites associated with risk of type 2 diabetes in two prospective cohorts of Chinese adults. Int J Epidemiol.2016; 45: 1507-16.

21. An D, Oh SF, Olszak T, Neves JF, Avci FY, Erturk-Hasdemir D *et al*. Sphingolipids from a symbiotic microbe regulate homeostasis of host intestinal natural killer T cells. *Cell* 2014;**156**: 123-33.

22. Yore MM, Syed I, Moraes-Vieira PM, Zhang T, Herman MA, Homan EA *et al*. Discovery of a class of endogenous mammalian lipids with anti-diabetic and anti-inflammatory effects. *Cell* 2014;**159**: 318-32.

23. Becker K, Frieling T, Saleh A, Haussinger D. Resolution of hydatid liver cyst by spontaneous rupture into the biliary tract. *J Hepatol* 1997;**26**: 1408-12.

24. Gustafson DL, Coulson AL, Feng L, Pott WA, Thomas RS, Chubb LS *et al*. Use of a medium-term liver focus bioassay to assess the hepatocarcinogenicity of 1,2,4,5-tetrachlorobenzene and 1,4-dichlorobenzene. *Cancer Lett* 1998; **129**: 39-44.

25. Hamaguchi M, Kojima T, Itoh Y, Harano Y, Fujii K, Nakajima T *et al*. The severity of ultrasonographic findings in nonalcoholic fatty liver disease reflects the metabolic syndrome and visceral fat accumulation. *Am J Gastroenterol* 2007; **102**: 2708-15.

26. Baba M, Furuya K, Bandou H, et al. Discrimination of individuals in a general population at high-risk for alcoholic and non-alcoholic fatty liver disease based on liver stiffness: a cross section study. *BMC Gastroenterol* 2011;**11**:70

27. Younossi ZM, Koenig AB, Abdelatif D, et al. Global epidemiology of nonalcoholic fatty liver disease—Meta‐analytic assessment of prevalence, incidence, and outcomes. *Hepatology* 2016;**64**:73-84

28 Bansal SK, Saxena V, Kandpal SuD, Gray WK, Walker RW, Goel D. The prevalence of hypertension and hypertension risk factors in a rural Indian community: A prospective door-to-door study. *J Cardiovasc Dis Res* 2012;**3**: 117-23.

29. Boussageon R, Supper I, Bejan-Angoulvant T, Kellou N, Cucherat M, Boissel JP *et al*. Reappraisal of metformin efficacy in the treatment of type 2 diabetes: a meta-analysis of randomised controlled trials. *PLoS Med* 2012; **9**:e1001204.

30. Nordestgaard BG, Palmer TM, Benn M, Zacho J, Tybjærg-Hansen A, Davey Smith G *et al*. The effect of elevated body mass index on ischemic heart disease risk: causal estimates from a mendelian randomisation approach. *PLoS Med* 2012; **9**:e1001212.

31. Fan N, Zhang L, Xia Z, Peng L, Wang Y, Peng Y. Sex-specific association between serum uric acid and nonalcoholic fatty liver disease in type 2 diabetic patients. *J Diabetes Res* 2016; **2016**: 3805372.

32. Yang Y, Zhou Y, Cheng S, Sun JL, Yao H, Ma L. Effect of uric acid on mitochondrial function and oxidative stress in hepatocytes. *Genet Mol Res* 2016; **15**: 15028644.

33. Li YL, Xie H, Musha H, Xing Y, Mei CX, Wang HJ *et al*. The risk factor analysis for type 2 diabetes mellitus patients with nonalcoholic fatty liver disease and positive correlation with serum uric acid. *Cell Biochem Biophys* 2015; **72**: 643-7.

For peer review only

34. Liu Z, Que S, Zhou L, Zheng S. Dose-response relationship of serum uric acid with metabolic syndrome and non-alcoholic fatty liver disease incidence: a meta-analysis of prospective studies. *Sci Rep* 2015; **5**: 14325.

35 Cai W, Wu X, Zhang B, et al. Serum uric acid levels and non-alcoholic fatty liver disease in Uyghur and Han ethnic groups in northwestern China. *Arq Bras Endocrinol Metabol* 2013;**57**:617-22.

36. Zhou Y, Wei F, Fan Y. High serum uric acid and risk of nonalcoholic fatty liver disease: A systematic review and meta-analysis. *Clin Biochem* 2016; **49**: 636 ‑ 42.

37 Parsons WH, Kolar MJ, Kamat SS, et al. AIG1 and ADTRP are atypical integral membrane hydrolases that degrade bioactive FAHFAs. *Nat Chem Biol* 2016;**12**:367-72.

38. Kolar MJ, Kamat SS, Parsons WH, et al. Branched Fatty Acid Esters of Hydroxy Fatty Acids Are Preferred Substrates of the MODY8 Protein Carboxyl Ester Lipase. *Biochemistry* 2016;**55**:4636-41.

39. Moraes-Vieira PM, Saghatelian A, Kahn BB. GLUT4 Expression in Adipocytes Regulates De Novo Lipogenesis and Levels of a Novel Class of Lipids With Antidiabetic and Anti-inflammatory Effects. *DIABETES* 2016;**65**:1808-15.

40. Zhu Q-F, Yan J-W, Gao Y, et al. Highly sensitive determination of fatty acid esters of hydroxyl fatty acids by liquid chromatography-mass spectrometry. *J Chromatogr B* 2017;**1061-1062**:34-40.

41. Lee J, Moraes-Vieira PM, Castoldi A, et al. Branched Fatty Acid Esters of Hydroxy Fatty Acids (FAHFAs) Protect against Colitis by Regulating Gut Innate and Adaptive Immune Responses. *J Biol Chem* 2016;**291**:22207-17.

42. Sitthi-amorn C, Poshyachinda V. Bias. *Lancet*. 1993;**342**: 286-8.

For peer review only

Table 1 Participant characteristics.

a. Subset

Indicators	Total	NAFLD	No NAFLD	*P* value
**n**	447	102 (22.8%)	345 (77.2%)	
**Age (years)**	44.3 ±11.9	44.6 ±10.8	44.0 ±12.1	0.609
**Age stage**	4.0±1.2	4.0±1.2	3.9±1.2	0.608
**Obesity**	235 (51.8%)	94	141	<0.001
**Body fat**	309 (68.1%)	89	220	<0.001
**Blood lipids**	87 (19.2%)	21	66	0.745
**Hypertension**	55 (12.1%)	23	32	0.003
**Male**	171 (38.3%)	64	107	<0.001
**Elevated liver enzymes**	18 (4.0%)	7	11	0.174

Age stages of 20-30, 30-40, 40-50, 50-60, ≥60 are 2, 3, 4, 5, 6.

b Total study population

Indicators	Total	NAFLD	No NAFLD	*P* value
**n**	1479	365 (24.7%)	1114 (75.3%)	
**Obesity**	809 (54.7%)	334	475	<0.001
**Body fat**	1003 (67.8%)	309	694	<0.001

For peer review only

**Blood lipid**	458 (30.9%)	163	295	<0.001
**High blood glucose**	71 (4.8%)	43	28	<0.001
**Hypertension**	314 (21.2%)	129	185	<0.001
**Abnormal uric acid**	83 (5.6%)	38	45	<0.001
**Male**	590 (39.8%)	214	376	<0.001
**Impaired liver function**	32 (0.2%)	12	20	0.142
**Elevated liver enzymes**	110 (0.7%)	53	57	<0.001

Conditions were defined as follows: obesity (BMI ≥ 24 kg/m^2), hypertension (blood pressure ≥ 140/90 mmHg or antihypertensive drug treatment), Body fat ratio ≥ 25 for women or ≥ 20 for men, blood triglycerides ≥ 1.7 mmol/L, blood fasting glucose ≥ 5.6 mmol/L, impaired liver function (positive HBsAg), liver enzyme elevation (AST ≥ 40 U/L or ALT ≥ 40 U/L), uric acid ≥ 357 μmol/L for women or ≥ 416 μmol/L for men.

Mean values are provided with standard deviation, unless otherwise noted as n (%). Differences between participants with and without NAFLD were evaluated with t-tests or the Wilcoxon-Mann-Whitney test for continuous variables and the chi-squared test for categorical variables. NAFLD, non-alcoholic fatty liver disease.

For peer review only

Table 2. NAFLD prevalence across age range.

**Age range (years)**	**Participants, n**	**Participants with NAFLD, n**	**NAFLD prevalence,%**	**U-test**
20-30	64	10	15.6	1.22
30-40	102	27	26.4	0.75
40-50	120	26	21.7	0.18
50-60	111	30	27.0	1.00
≥60	50	9	18.0	0.64
Total	447	102	22.8	1.64 (α=0.1)

NAFLD, non-alcoholic fatty liver disease.

For peer review only

Table 3. Multivariate adjusted models for liver disease subtypes.

a Subset

Variables	OR	*p* value
Sex (male)	3.484	<0.001
Obesity	11.738	<0.001
Body fat	2.285	0.033
Elevated liver enzyme	2.237	0.105
Hypertension	1.865	0.089

b The whole

Variables	OR	*p* value
Sex (male)	2.646	<0.001
Obesity	8.494	<0.001
Body fat	1.833	0.001
Blood lipid	1.340	0.046
Blood glucose	3.324	<0.001
Impaired liver function	1.859	0.094
Elevated liver enzyme	3.150	<0.001
Hypertension	1.451	0.017

Conditions were defined as follows: female sex=1, obesity (BMI ≥ 28 kg/m^2), hypertension (blood pressure ≥ 140/90 mmHg or antihypertensive drug treatment), body fat ratio ≥ 25 for women or ≥ 20 for men, blood fasting

For peer review only

glucose ≥ 6.1 mmol/L, liver enzyme elevation (AST ≥ 40 U/L or ALT ≥ 40 U/L)

NAFLD, non-alcoholic fatty liver disease.

Odds ratios and the corresponding p values derived from binary or multiple logistic regression analyses using SPSS software version 17.0 (SPSS Inc., Chicago, IL, USA)

For peer review only

Table 4. Metabolites associated with non-alcoholic fatty liver disease.

**Metabolite**	**OR, *P* value**	**AUC, *P* value**
**Uric acid**	2.755 <0.001	0.579 <0.001
**Sphingosine**	1.448 0.156	0.489 0.760
**OAHOA**	1.340 0.046	0.612 0.001

Conditions were defined: Sphingosine < 2 nmol/L, OAHOA (oleic acid -hydroxy oleic acid) < 5 nmol/L.

NAFLD, non-alcoholic fatty liver disease.

Odds ratios (OR), area under curve (AUC) and the corresponding p values derived from binary or multiple logistic regression, or ROC analyses using SPSS software version 17.0 (SPSS Inc., Chicago, IL, USA)

For peer review only

STROBE Statement—checklist of items that should be included in reports of observational studies

	Item No.	Recommendation	Page No.
**Title and abstract**	1	(*a*) Indicate the study's design with a commonly used term in the title or the abstract	1
		(*b*) Provide in the abstract an informative and balanced summary of what was done and what was found	2
**Introduction**			
Background/rationale	2	Explain the scientific background and rationale for the investigation being reported	4
Objectives	3	State specific objectives, including any prespecified hypotheses	5
**Methods**			
Study design	4	Present key elements of study design early in the paper	5
Setting	5	Describe the setting, locations, and relevant dates, including periods of recruitment, exposure, follow-up, and data collection	5,6,7,8
Participants	6	(*a*) *Cohort study*—Give the eligibility criteria, and the sources and methods of selection of participants. Describe methods of follow-up<br>*Case-control study*—Give the eligibility criteria, and the sources and methods of case ascertainment and control selection. Give the rationale for the choice of cases and controls<br>*Cross-sectional study*—Give the eligibility criteria, and the sources and methods of selection of participants	5,6
		(*b*)*Cohort study*—For matched studies, give matching criteria and number of exposed and unexposed<br>*Case-control study*—For matched studies, give matching criteria and the number of controls per case	
Variables	7	Clearly define all outcomes, exposures, predictors, potential confounders, and effect modifiers. Give diagnostic criteria, if applicable	6,7,8
Data sources/ measurement	8*	For each variable of interest, give sources of data and details of methods of assessment (measurement). Describe comparability of assessment methods if there is more than one group	6,7,8
Bias	9	Describe any efforts to address potential sources of bias	6
Study size	10	Explain how the study size was arrived at	5

Continued on next page

For peer review only

Quantitative variables	11	Explain how quantitative variables were handled in the analyses. If applicable, describe which groupings were chosen and why	7,8
Statistical methods	12	(*a*) Describe all statistical methods, including those used to control for confounding	8
		(*b*) Describe any methods used to examine subgroups and interactions	8
		(*c*) Explain how missing data were addressed	
		(*d*) *Cohort study*—If applicable, explain how loss to follow-up was addressed<br>*Case-control study*—If applicable, explain how matching of cases and controls was addressed<br>*Cross-sectional study*—If applicable, describe analytical methods taking account of sampling strategy	8
		(*e*) Describe any sensitivity analyses	
**Results**			
Participants	13*	(a) Report numbers of individuals at each stage of study—eg numbers potentially eligible, examined for eligibility, confirmed eligible, included in the study, completing follow-up, and analysed	8,9
		(b) Give reasons for non-participation at each stage	
		(c) Consider use of a flow diagram	
Descriptive data	14*	(a) Give characteristics of study participants (eg demographic, clinical, social) and information on exposures and potential confounders	8,9
		(b) Indicate number of participants with missing data for each variable of interest	
		(c) *Cohort study*—Summarise follow-up time (eg, average and total amount)	
Outcome data	15*	*Cohort study*—Report numbers of outcome events or summary measures over time	
		Case-control study—Report numbers in each exposure category, or summary measures of exposure	
		Cross-sectional study—Report numbers of outcome events or summary measures	8,9
Main results	16	(*a*) Give unadjusted estimates and, if applicable, confounder-adjusted estimates and their precision (eg, 95% confidence interval). Make clear which confounders were adjusted for and why they were included	9,10,11
		(*b*) Report category boundaries when continuous variables were categorized	
		(*c*) If relevant, consider translating estimates of relative risk into absolute risk for a meaningful time period	

Continued on next page

For peer review only

For peer review only - http://bmjopen.bmj.com/site/about/guidelines.xhtml

Other analyses	17	Report other analyses done—eg analyses of subgroups and interactions, and sensitivity analyses	
**Discussion**			
Key results	18	Summarise key results with reference to study objectives	11,12,13
Limitations	19	Discuss limitations of the study, taking into account sources of potential bias or imprecision. Discuss both direction and magnitude of any potential bias	14
Interpretation	20	Give a cautious overall interpretation of results considering objectives, limitations, multiplicity of analyses, results from similar studies, and other relevant evidence	11,12,13
Generalisability	21	Discuss the generalisability (external validity) of the study results	14
**Other information**			
Funding	22	Give the source of funding and the role of the funders for the present study and, if applicable, for the original study on which the present article is based	15

*Give information separately for cases and controls in case-control studies and, if applicable, for exposed and unexposed groups in cohort and cross-sectional studies.

Note: An Explanation and Elaboration article discusses each checklist item and gives methodological background and published examples of transparent reporting. The STROBE checklist is best used in conjunction with this article (freely available on the Web sites of PLoS Medicine at http://www.plosmedicine.org/, Annals of Internal Medicine at http://www.annals.org/, and Epidemiology at http://www.epidem.com/). Information on the STROBE Initiative is available at www.strobe-statement.org.

For peer review only

For peer review only - http://bmjopen.bmj.com/site/about/guidelines.xhtml

Risk Factors and Biomarkers of Non-alcoholic Fatty Liver Disease: an observational cross-sectional population survey

Journal:	*BMJ Open*
Manuscript ID	bmjopen-2017-019974.R3
Article Type:	Research
Date Submitted by the Author:	14-Feb-2018
Complete List of Authors:	Hu, Xiao-Yu ; Wuhan Polytechnic University, School of Biology and Pharmaceutical Engineering<br>Li, Yun ; Wuhan Polytechnic University, School of Biology and Pharmaceutical Engineering<br>Li, Long-Quan ; Wuhan Polytechnic University, , School of Biology and Pharmaceutical Engineering<br>Zheng, Yuan ; Wuhan Polytechnic University, , School of Biology and Pharmaceutical Engineering<br>Lv, Jia-Hong ; Wuhan Polytechnic University, , School of Biology and Pharmaceutical Engineering<br>Huang , Shu-Chun; Wuhan Polytechnic University, School of Biology and Pharmaceutical Engineering<br>Zhang, Wei-Nong ; Wuhan Polytechnic University, School of Biology and Pharmaceutical Engineering<br>Liu, Liang; Wuhan Polytechnic University, School of Biology and Pharmaceutical Engineering<br>Zhao, Ling; Wuhan Polytechnic University, School of Biology and Pharmaceutical Engineering<br>Liu, Zhiguo; Wuhan Polytechnic University, School of Biology and Pharmaceutical Engineering<br>Zhao, Xiu-Ju
**Primary Subject Heading**:	Public health
Secondary Subject Heading:	Gastroenterology and hepatology, Epidemiology
Keywords:	EPIDEMIOLOGY, Hepatology < INTERNAL MEDICINE, PUBLIC HEALTH

Risk Factors and Biomarkers of Non-alcoholic Fatty Liver Disease: an observational cross-sectional population survey

Xiao-Yu Hu[1], Yun Li[1], Long-Quan Li[1], Yuan Zheng[1], Jia-Hong Lv[1], Shu-Chun Huang[1], Weinong Zhang[1], Liang Liu[1], Ling Zhao[1], Zhuiguo Liu[1], Xiu-Ju Zhao[1,2,3*]

1 School of Biology and Pharmaceutical Engineering, Wuhan Polytechnic University, 68 South Xuefu Road, Changqing Garden, Wuhan, Hubei 430023, P. R. China

2 Hubei Key Laboratory of Lipid Chemistry and Nutrition of Oil, Oil Crops Research Institute of the Chinese Academy of Agricultural Sciences, Wuhan, Hubei 430062, P. R. China

3 Department of Nutrition and Food Science, Texas A&M University, College Station, TX 77843, U.S.A.

Corresponding

Xiu-Ju Zhao

E-mail: dzrdez@163.com, Tel. and Fax: +862783956793, School of Biology and Pharmaceutical Engineering, Wuhan Polytechnic University, 68 South Xuefu Road, Changqing Garden, Wuhan 430023, P. R. China

Abbreviations: ALT, alanine aminotransferase; AST, aspartate aminotransferasek; BFR, body fat ratio; BMI, body mass index; HBsAg, hepatitis B surface antigen; NAFLD, non-alcoholic fatty liver disease.

For peer review only

ABSTRACT

Objectives Non-alcoholic fatty liver disease (NAFLD) is a major public health burden in China and its prevalence is increasing. This study aimed to determine risk factors and biomarkers of NAFLD.

Design An observational cross-sectional primary survey.

Setting Central China.

Participants 1479 aged over 18 and below 80 years, not currently being treated for cancer or infectious disease or no surgery in the previous year, and no previous history of cancer or an infectious disease. Participants underwent clinical examination, metabolomic assay, and anthropometric assessment. Univariate and logistic regression analyses were used to evaluate associations between covariates and NAFLD.

Main outcome measures Risk factors and metabolic biomarkers including sex, body mass index, hypertension, body fat ratio, blood triglycerides, blood fasting glucose, liver enzyme elevation, uric acid, and oleic acid-hydroxy oleic acid.

Results Data from the 447 participants (mean age 44.3 ± 11.9 years) were analyzed. And the prevalence of NAFLD was 24.7%. Male (OR 3.484; 95% CI 2.028–5.988), body mass index ≥ 24 kg/m^2 (OR 8.494; 95% CI 5.581–12.928), body fat ratio (≥ 25, women; ≥ 20, men) (OR 1.833; 95% CI 1.286–2.756), triglycerides ≥ 1.7 mmol/L (OR 1.340; 95% CI 1.006–1.785), fasting glucose ≥ 6.1 mmol/L (OR 3.324; 95% CI 1.888–5.850), blood pressure ≥ 140/90 mmHg or antihypertensive drug treatment (OR 1.451, 95% CI 1.069–1.970), uric acid (≥ 357 μmol/L, women; ≥ 416 μmol/L, men) (OR 2.755, 95% CI 2.009–3.778), and oleic acid-hydroxy oleic acid (OAHOA < 5 nmol/L) (OR 1.340, 95% CI 1.006-1.785) were independent predictors of NAFLD (all $p < 0.05$). These results were verified by the whole 1479 participants.

Conclusions NAFLD was common among the study participants. In particular,

For peer review only

NAFLD was correlated with uric acid. We identified OAHOA as a novel marker of NAFLD prevalence. It provides a reference on the prevention of NAFLD and related metabolic diseases with the rapid urbanization, technological advancement, and population aging in China over the recent decades.

Strengths and limitations of this study

Risk factors of NAFLD in central China.

Biomarkers of NAFLD using metabolomics.

Prediction ability of metabolic markers using ROC.

No information on education.

Keywords: uric acid; oleic acid-hydroxy oleic acid; male; non-alcoholic fatty liver disease (NAFLD)

For peer review only

INTRODUCTION

China has the world's largest population and is undergoing rapid economic growth and social reform. This advancement has paralleled demographic, lifestyle, and cultural changes that have exerted notable effects on the health profile of China's residents and placed significant constraints on the country's healthcare system. [1]

Such changes are apparent in major cities of central China, such as Wuhan. The prevalence of non-alcoholic fatty liver disease (NAFLD) is increasing, now is the most common chronic disease among Chinese adults in China, and the rates exceed those of equally important epidemics including obesity, hypertension, and type II diabetes. NAFLD is followed by an increase in overall cardiovascular morbidity and mortality.[2-4] It has been estimated that NAFLD will be the most frequent indicator for liver transplantation and regeneration in the coming decades.[5-7] Additionally, because population aging and the continual increase in obesity and hypertension rates with reforming and open-up over the recent decades, the prevalence and impact of NAFLD in China is expected to increase. Accordingly, NAFLD is a major public health burden.

The reported prevalence of NAFLD among Chinese adults ranges from 15% to 30%.[5-7] NAFLD prevalence increases with age, most notably from the fourth decade of life onward (40 - 60 years of age).[5,6,8,9] Because of endocrine function variations and fat redistribution, the prevalence and risk factors for NAFLD may vary across different ages and between male and female populations.[10-13] However, the association of age and sex with NAFLD in central China is still unclear. Furthermore, the association of hypertension, obesity, hyperlipidemia, insulin resistance, and

For peer review only

diabetes with NAFLD has been widely evaluated in adult cohorts [14-16], and these metabolic traits are well-accepted risk factors at present for NAFLD. Nonetheless, limited data are available relating to the association of NAFLD with other covariant traits [17,18]. Metabolomics provides the integrated information on biological status and can investigate the molecular variations with disease phenotyping[19,20]; several markers for NAFLD have been identified previously[5,6]. Sphingolipids (SLs) and branched fatty acid esters of hydroxy fatty acids (FAHFAs) have reported to protect against autoimmune and allergic disorders and type 2 diabetes, respectively[21,22]; the effects of SLs and FAHFAs on NAFLD are largely unclear.

Our present study was aimed at investigating the prevalence of NAFLD, elucidating the association of NAFLD with age, sex, metabolic factors, and markers of liver injury, and identifying novel metabolites associated with NAFLD risk, via clinical examination, anthropometric assessment, and targeted metabolomics.

METHODS

Study population

The ethics committee of Wuhan Union Hospital approved the study, and all participants provided written informed consent prior to enrolment. The Wuhan Study is an observational cross-sectional study of participants recruited from the Wuhan Municipality, central China. Participants were recruited from routine health checkups to assess the utility of health checkups in determining the prevalence of and risk factors for NAFLD. Each participant completed a comprehensive interview and clinical examination that involved the collection of fasting blood and urine samples,

For peer review only

abdominal ultrasonography, and anthropometric assessment.

Interview

The interview, followed by the clinical examination, was performed by a physician and was designed to obtain information concerning demographic characteristics, medical history, and comorbid conditions. Participants aged over 18 and below 80 years were included if they were not currently being treated for cancer or infectious disease or had undergone surgery in the previous year, and if they had no previous history of cancer or an infectious disease.

Biochemistry

Fasting blood and urine samples were collected on the clinical examination. Blood alanine aminotransferase (ALT), alkaline phosphatase, aspartate aminotransferase (AST), gamma-glutamyl transferase, glucose, total bilirubin, triglycerides, and uric acid levels were measured using automatic enzymatic procedures (Nanjing Jiancheng Sci-tech, China). Hepatitis B surface antigen (HBsAg), triiodothyronine, thyroxine (T4), and thyroid-stimulating hormone were examined via automatic immunoassays (Roche Diagnostics GmbH, Mannheim, DE).

Diagnosis of NAFLD

Abdominal ultrasonography was performed on all participants by accredited medical technicians using a Hitachi HI VISION 900 ultrasound machine. Images were evaluated by a senior pathologist. NAFLD was diagnosed by the technician according to ultrasonography [23-25]. Participants with any of the following possible secondary

For peer review only

causes of fatty liver were excluded from the current analyses:

(1) excessive alcohol intakes[26], (2) anti-HCV or positive HBsAg, or (3) use of drugs historically for treating fatty liver (i.e., amiodarone, corticosteroids, methotrexate, or tamoxifen) [13].

Metabolic covariates

Anthropometric measurements were performed by accredited nurses. Body mass index (BMI) was defined as measured weight (kg) divided by height squared (m^2). Body fat ratio (BFR) was equal to fat weight (kg) divided by body weight (kg). The average of two resting blood pressure measurements at a single visit after a 10-min break, was used for analysis.

Metabolic traits were defined: obesity (BMI ≥ 28 kg/m^2), hypertension (blood pressure ≥ 140/90 mmHg or antihypertensive drug treatment), BFR ≥ 25 for women or ≥ 20 for men, blood triglycerides ≥ 1.7 mmol/L, blood fasting glucose ≥ 6.1 mmol/L, liver enzyme elevation (AST ≥ 40 U/L or ALT ≥ 40 U/L), uric acid ≥ 357 μmol/L for women or ≥ 416 μmol/L for men, or impaired OAHOA(< 5 nmol/L).

Targeted LC/MS analysis of SLs and FAHFAs

To identify SLs[21], silica column-affectionate lipid fractions were analyzed by reverse-phase HPLC-MS/MS (Agilent C18 column connected with Thermo Scientific LTQXL; gradients of water to methanol, 10:90 to 0:100) to identify spots present in both control and LD. Identified lipid species of interest were purified by reverse-phase HPLC (Varian Prostar/Agilent C18 column) and subjected to structural analysis.

For peer review only

FAHFAs were measured by HPLC-MS [22] (an Agilent 6410 Triple Quad LC/MS via MRM in a negative ionization mode). Briefly, extracted and fractionated samples were resolved in 25 ml MeOH; 10 ml was injected for further analysis. A Luna C18(2) (Phenomenex) column was used with an in-line filter (Phenomenex). Distinct FAHFAs were resolved via isocratic flow (0.2 ml/min for 120 min, solvent: 93:7 MeOH:H_2O, 5 mM ammonium acetate, 0.01% ammonium hydroxide). Transitions for OAHOAs were m/z 561.5 → m/z 281.2 (Collision Energy [CE] = 30 V), and m/z 561.5 → m/z 279.2 (CE = 25 V).

STATISTICAL ANALYSES

Baseline characteristics were analyzed using descriptive statistics. U-tests, chi-square tests, or Wilcoxon rank-sum tests (for medians) and Student's t-tests (for means) were used to evaluate the significance of differences in the distribution of categorical data and continuous data, respectively. To examine relations between traits and NAFLD, we performed binary or multiple logistic regression analyses. We calculated the area under the receiver operating curve (AUC) to assess prediction ability of metabolic markers. A *p*-value of < 0.05 was considered statistically significant unless stated otherwise. Data analyses were performed using SPSS (v17.0, SPSS Inc., Chicago, IL, USA).

RESULTS

Study population

In total, 1500 participants were enrolled. Twenty-one participants were excluded due to the absence of clinical data. Thus, a total of 1479 study participants were

For peer review only

included in the final analysis, of which 447 reported their age and had no more than one disease, if any (denoted as the "subset"). Patients' general characteristics are shown in Table 1. Men accounted for 40% of the study population (38% of the subset). Participants' mean age was 44.3 ± 11.9 (range 20–74) years.

Prevalence of NAFLD

The prevalence of NAFLD, as determined by ultrasound and biopsy, was 24.7%, (22.8% in the subset).

The peak prevalence of NAFLD was 26.4% and 26.3% in this study between the ages of 30–40 years and 50–60 years, respectively (Table 2).

The prevalence of NAFLD demonstrated an increasing trend with advancing age (OR 1.049, p = 0.607), and after adjustment for sex and metabolic features, NAFLD tended to be inversely correlated with age (OR 0.844; 95% CI 0.667–1.068; p = 0.157). NAFLD was diagnosed in 37.4% of men and 13.8% of women (p = 0.084). In multivariate analysis, after adjustment for metabolic features and age, male sex was associated with NAFLD (OR 3.484; 95% CI 2.028–5.988; p < 0.001, Table 3a).

Association between NAFLD and metabolic features

Among metabolic covariates, obesity and hypertension occurred more frequently in participants with advancing age (OR 1.471 and 1.822, respectively; p < 0.001). Increased body fat was more prevalent in women than in men (OR 2.042; p < 0.001; [OR 1.754; p = 0.007 in the subset]), and obesity (OR 0.537, p < 0.001), increased blood lipids (OR 0.829, p < 0.101), and hypertension (OR 0.769; p < 0.041) were more prevalent in men than in women (OR 0.472, p <0.001; OR 0.902, p < 0.673; and

OR 0.602; p < 0.080, respectively, in the subset).

In univariate analysis, all metabolic and anthropometric traits were significantly associated with NAFLD. In logistic regression analysis, after adjustment for sex, BMI ≥ 24 kg/m^2 (OR 8.494; 95% CI 5.581–12.928; $p < 0.001$), BFR ≥ 25 for women and ≥ 20 for men (OR 1.833; 95% CI 1.286–2.756; $p = 0.001$), triglycerides ≥ 1.7 mmol/L (OR 1.340; 95% CI 1.006–1.785; $p = 0.046$), fasting glucose ≥ 6.1 mmol/L (OR 3.324; 95% CI 1.888–5.850; $p < 0.001$), blood pressure (BP)≥ 140/90 mmHg or antihypertensive drug treatment (OR 1.451, 95% CI 1.069–1.970; $p = 0.017$), uric acid ≥ 357 μmol/L for women and ≥ 416 μmol/L for men (OR 2.755, 95% CI 2.009–3.778; $p < 0.001$), and total OAHOA < 5 nmol/L (OR 1.340, 95% CI 1.006-1.785; $p = 0.046$) [after adjustment for age stages and sex in the subset, ORs for BMI, BFR, and BP: 11.738 (95% CI 5.193–26.530; $p < 0.001$), 2.285 (95% CI 1.067–4.893; $p = 0.033$) and 1.865 (95% CI 0.910–3.824; $p = 0.089$), respectively] were independent predictors of NAFLD (Table 3a, b and Table 4). The prevalence of NAFLD demonstrated an increasing trend with lowering sphingosines (OR 1.448, $p = 0.156$; Table 4). Substitution of age ranges for continuous data (i.e., absolute age) in logistic regression did not modify the relationships. When each metabolic trait was analyzed independently, after adjustment for age and sex, ORs increased for obesity and hypertension with increasing age, and ORs increased for BFR with increasing age for patients aged <50 years and decreased for patients aged ≥50 years. However, significant interactions were only observed between age and obesity ($p < 0.001$), and between age and hypertension ($p < 0.001$).

Association between NAFLD and liver enzymes

For peer review only - http://bmjopen.bmj.com/site/about/guidelines.xhtml

Participants with NAFLD had higher liver enzyme levels than participants without this condition ($p < 0.001$). Abnormal liver enzymes were also significantly associated with NAFLD, independent of sex (OR 3.150, $p < 0.001$). Normal liver enzyme levels, defined according to local guidelines (ALT <40 U/L), were found in 85% of participants with NAFLD.

DISCUSSION

Our results demonstrated that NAFLD was strong associated with metabolic traits including higher body fat, obesity, hyperlipidemia, and impaired fasting glucose. We observed a higher prevalence of NAFLD in men than in women. We conformed the association of impaired uric acid, and abnormal liver enzymes with NAFLD; and further identified impaired total OAHOA as a novel biomarker of NAFLD prevalence.

The average and peak prevalence of NAFLD in this study was 24.7 and more than 26%, respectively, close to the global prevalence of NAFLD, which was 25.24% with highest prevalence in the Middle East(31.79%) and South America(30.45%), and lowest in Africa(13.48%)[27]. The average and peak prevalence of NAFLD in Shanghai [5] was 20.82% and 28.44%, respectively, and those in Guangdong [6] was 17.2% and 27.4%, respectively. The former study was conducted from 2002 to 2003, and the latter in 2005. Our study, performed in 2010, revealed that the average prevalence is increasing, and the peak prevalence occurred at a younger age (30-40 years) than was reported by the previous studies in Shanghai (age of 60-69) [5], Guangdong (age of 60-69) [6] and worldwide (mean age of 70-79)[27]. Thus, more

For peer review only

attention should be given to the health of people in their 30s, when many marry and adopt lifestyle changes, especially in urban China. Unfortunately, we did not have access to information on participants' activity levels, and thus, the effects of physical activity on NAFLD were not assessed. The reasons underlying the observed earlier peak prevalence require further investigation.

We observed that male sex was a risk factor for NAFLD. This finding differs from the results of the Guangdong study [6], and the Rotterdam study [13]. However, this finding is similar to that of the Shanghai study.

There are several possibilities for this discrepancy. First, the observed sex difference in NAFLD prevalence may be the result of body fat, which is a risk factor for NAFLD and was higher in male than in female in this study. Therefore, male sex may promote NAFLD through mediators such as increased body fat and hormonal change. Second, the sex difference in NAFLD may also result from a lower prevalence of boosted serum triglycerides, glucose, and hypertension in females compared with males. A lower prevalence of boosted serum triglycerides, glucose and hypertension in females was reported in a previous study [28]. However, a causal relationship between NAFLD and these serum metabolic markers, as suggested by previous research [29,30], could not be established in our research, due to its cross-sectional characteristics. Third, abnormal liver enzymes were more prevalent in men than in women, and both were independent predictors of NAFLD.

We observed that the associations of the identified metabolites with NAFLD risk were pronounced in the Wuhan cohort. These metabolites might play a major role in the development of onset NAFLD. Uric acid was particularly associated with NAFLD in men with type 2 diabetes, besides insulin resistance and other metabolic factors

For peer review only

[31]. High concentrations of uric acid induce the accumulation of reactive oxygen species in hepatocyte mitochondria, ultimately leading to mitochondrial damage [32], and uric acid was positively correlated with other metabolic traits and was a risk factor for type 2 diabetes mellitus with NAFLD [33].

Serum uric acid (SUA) is produced by purine nucleotide catabolism.[34] The SUA subjects have an about 2-fold higher risk of NAFLD as shown by ultrasonography. The risk may be independent of age, sex, and obesity (indicated by BMI and waist circumference). SUA is an independent risk factor in NAFLD in both Uyghurs and Hans in northwestern China.[35] Among participants with elevated SUA levels, female likely showed a greater risk of NAFLD than male.[36]

OAHOAs are present in humans, which are an endogenous branched fatty acid esters of hydroxy fatty acids (FAHFAs) and levels are reduced with obesity and insulin resistance. OAHOA levels in serum are correlated highly with whole-body insulin sensitivity. OAHOAs are endogenous GPR120 ligands and may also exert anti-inflammatory effects in vivo through fatty acid-activated GPCRs, such as GPR120. AIG1 and ADTRP, atypical integral membrane hydrolases degrade bioactive FAHFAs,[37] and branched FAHFAs are preferred substrates of MODY8, a protein carboxyl ester lipase.[38] Changes in the concentrations of these anti-inflammatory metabolites and in their signaling networks may provide new targets for metabolic and inflammatory diseases [22]. GLUT4 expression and levels of FAHFAs with antidiabetic and anti-inflammatory effects regulates de novo lipogenesis in adipocytes.[39] Compared to healthy controls, FAHFAs significantly decreased in sera of breast cancer patients.[40] Branched FAHFAs protect against colitis by controlling gut innate and adaptive immune responses.[41]

For peer review only

Our study also has some potential limitations. First, we cannot rule out the possibility of biased selection, since non-responders may have had different morbidities. Volunteers in research studies tend to be better educated, healthier, and have better lifestyles [42]. Consequently, estimations of the prevalence of NAFLD may have been biased. Second, only 447 of the 1479 participants provided information on their ages, and we did not obtain data on participants' educational levels or incomes. Third, specific OAHOA isomers [22] require further study.

CONCLUSIONS

NAFLD is common in the study population. In this observational cross-sectional primary survey, we found that NAFLD was associated with male sex, and the peak prevalence occurred at a younger age. In particular, NAFLD was correlated with uric acid. Moreover, we identified OAHOA as a novel biomarker of NAFLD. Further studies are needed to explore the potential factors contributing to these relationships. However, this study is valuable in that it provides a reference on the prevention of NAFLD and related metabolic diseases with the rapid urbanization, technological advancement, and population aging in China.

Contributors Designed the study: Xiu-Ju Zhao. Analyzed data: Xiao-Yu Hu, Yun Li, Long-Quan Li, Yuan Zheng, Jia-Hong Lv, Shu-Chun Huang, Xiu-Ju Zhao. Wrote

For peer review only

and critically review the manuscript: Xiao-Yu Hu, Weinong Zhang, Liang Liu, Ling Zhao, Zhuiguo Liu, Xiu-Ju Zhao.

Conflict of interest: There is no conflict of interest.

Ethics approval The ethics committee of Wuhan Union Hospital approved the study, and all participants provided written informed consent prior to enrolment.

Data sharing statement Data are available on request.

FUNDING STATEMENT: This work was supported in part by the Opening Project of Hubei Key Laboratory of Lipid Chemistry and Nutrition of Oil (201506), and National Nature Science Foundation of China (21602166). They had no role in this study.

For peer review only

REFERENCES

1. Yusuf S, Reddy S, Ounpuu S, Anand S. Global burden of cardiovascular diseases: part I: general considerations, the epidemiologic transition, risk factors, and impact of urbanization. *Circulation* 2001;**104**: 2746-53.
2. Dunn W, Xu R, Wingard D, Rogers C, Angulo P, Younossi Z *et al*. Suspected Nonalcoholic Fatty Liver Disease and Mortality Risk in a Population-Based Cohort Study. *Am J Gastroenterol* 2008;**103**: 2263-71.
3. Ong JP, Pitts A, Younossi ZM. Increased overall mortality and liver-related mortality in non-alcoholic fatty liver disease. *J Hepatol* 2008;**49**: 608-12.
4. Targher G., Day CP, Bonora E. Risk of cardiovascular disease in patients with nonalcoholic fatty liver disease. *N Engl J Med* 2010;**36**: 1341-50.
5. Fan JG, Zhu J, Li X J, Chen L, Li L, Dai F *et al*. Prevalence of and risk factors for fatty liver in a general population of Shanghai, China. *J Hepatol* 2005;**43**: 508-14.
6. Zhou YJ, Li YY, Nie YQ, Ma JX, Lu LG, Shi SL *et al*. Prevalence of fatty liver disease and its risk factors in the population of South China. *World J Gastroenterol* 2007;**13**: 6419-24.
7. Fan JG., Farrell GC. Epidemiology of non-alcoholic fatty liver disease in China. *J Hepatol* 2009;**50**: 204-10.
8. Schwimmer JB, Deutsch R, Kahen T, Lavine J E, Stanley C, Behling C. Prevalence of fatty liver in children and adolescents. *Pediatrics* 2006;**118**: 1388-93.
9. Ayonrinde OT, Olynyk JK, Beilin LJ, Mori TA, Pennell CE, de Klerk N *et al*. Gender-specific differences in adipose distribution and adipocytokines influence adolescent nonalcoholic fatty liver disease. *Hepatology* 2011;**53**: 800-9.
10. Roy AK, Chatterjee B. Sexual Dimorphism in the Liver. *Annu Rev Physiol* 1983;**45**: 37-50.
11. Sepe A, Tchkonia T, Thomou T, Zamboni M, Kirkland JL. Aging and regional differences in fat cell progenitors - a mini-review. *Gerontology* 2011;**57**: 66-75.
12. Takasugi M, Hayakawa K, Arai D, Shiota K. Age- and sex-dependent DNA hypomethylation controlled by growth hormone in mouse liver. *Mech Ageing Dev* 2013;**134**: 331-7.
13. Koehler EM, Schouten JN, Hansen BE, van Rooij FJ, Hofman A, Stricker BH, *et al*. Prevalence and risk factors of non-alcoholic fatty liver disease in the elderly: results from the Rotterdam study. *J Hepatol* 2012;**57**: 1305-11.
14. Marchesini G, Brizi M, Morselli-Labate A M, Bianchi G, Bugianesi E, McCullough AJ *et al*. Association of nonalcoholic fatty liver disease with insulin resistance. *Am J Med* 1999;**107**: 450-5.
15. Pagano G, Pacini G, Musso G, Gambino R, Mecca F, Depetris N *et al*. Nonalcoholic steatohepatitis, insulin resistance, and metabolic syndrome: further evidence for an etiologic association. *Hepatology* 2002;**35**: 367-72.
16. Cui J, Chen CH, Lo MT, Schork N, Bettencourt R, Gonzalez MP *et al*. Shared genetic effects between hepatic steatosis and fibrosis: A prospective twin study. *Hepatology* 2016; **64**: 1547–58.
17. Bano A, Chaker L, Plompen EPC, Hofman A, Dehghan A, Franco OH *et al*. Thyroid function and the risk of non-alcoholic fatty liver disease: The Rotterdam Study. *J Clin Endocrinol Metab*. 2016; **101**: 3204–11.
18. Ting WH, Chien MN, Lo FS, Wang CH, Huang CY, Lin CL *et al*. Association of cytotoxic

For peer review only

T-lymphocyte-associated protein 4 (CTLA4) gene polymorphisms with autoimmune thyroid disease in children and adults: case-control study. *PLoS One*. 2016; **11**:e0154394.

19. Menni C, Kastenmüller G, Petersen AK, Bell JT, Psatha M, Tsai PC *et al*. Metabolomic markers reveal novel pathways of ageing and early development in human populations. *Int J Epidemiol* 2013; **42**: 1111–9.

20. Qiu, G. et al. Plasma metabolomics identified novel metabolites associated with risk of type 2 diabetes in two prospective cohorts of Chinese adults. Int J Epidemiol.2016; 45: 1507-16.

21. An D, Oh SF, Olszak T, Neves JF, Avci FY, Erturk-Hasdemir D *et al*. Sphingolipids from a symbiotic microbe regulate homeostasis of host intestinal natural killer T cells. *Cell* 2014;**156**: 123-33.

22. Yore MM, Syed I, Moraes-Vieira PM, Zhang T, Herman MA, Homan EA *et al*. Discovery of a class of endogenous mammalian lipids with anti-diabetic and anti-inflammatory effects. *Cell* 2014;**159**: 318-32.

23. Becker K, Frieling T, Saleh A, Haussinger D. Resolution of hydatid liver cyst by spontaneous rupture into the biliary tract. *J Hepatol* 1997;**26**: 1408-12.

24. Gustafson DL, Coulson AL, Feng L, Pott WA, Thomas RS, Chubb LS *et al*. Use of a medium-term liver focus bioassay to assess the hepatocarcinogenicity of 1,2,4,5-tetrachlorobenzene and 1,4-dichlorobenzene. *Cancer Lett* 1998; **129**: 39-44.

25. Hamaguchi M, Kojima T, Itoh Y, Harano Y, Fujii K, Nakajima T *et al*. The severity of ultrasonographic findings in nonalcoholic fatty liver disease reflects the metabolic syndrome and visceral fat accumulation. *Am J Gastroenterol* 2007; **102**: 2708-15.

26. Baba M, Furuya K, Bandou H, et al. Discrimination of individuals in a general population at high-risk for alcoholic and non-alcoholic fatty liver disease based on liver stiffness: a cross section study. *BMC Gastroenterol* 2011;**11**:70

27. Younossi ZM, Koenig AB, Abdelatif D, et al. Global epidemiology of nonalcoholic fatty liver disease—Meta‐analytic assessment of prevalence, incidence, and outcomes. *Hepatology* 2016;**64**:73-84

28 Bansal SK, Saxena V, Kandpal SuD, Gray WK, Walker RW, Goel D. The prevalence of hypertension and hypertension risk factors in a rural Indian community: A prospective door-to-door study. *J Cardiovasc Dis Res* 2012;**3**: 117-23.

29. Boussageon R, Supper I, Bejan-Angoulvant T, Kellou N, Cucherat M, Boissel JP *et al*. Reappraisal of metformin efficacy in the treatment of type 2 diabetes: a meta-analysis of randomised controlled trials. *PLoS Med* 2012; **9**:e1001204.

30. Nordestgaard BG, Palmer TM, Benn M, Zacho J, Tybjærg-Hansen A, Davey Smith G *et al*. The effect of elevated body mass index on ischemic heart disease risk: causal estimates from a mendelian randomisation approach. *PLoS Med* 2012; **9**:e1001212.

31. Fan N, Zhang L, Xia Z, Peng L, Wang Y, Peng Y. Sex-specific association between serum uric acid and nonalcoholic fatty liver disease in type 2 diabetic patients. *J Diabetes Res* 2016; **2016**: 3805372.

32. Yang Y, Zhou Y, Cheng S, Sun JL, Yao H, Ma L. Effect of uric acid on mitochondrial function and oxidative stress in hepatocytes. *Genet Mol Res* 2016; **15**: 15028644.

33. Li YL, Xie H, Musha H, Xing Y, Mei CX, Wang HJ *et al*. The risk factor analysis for type 2 diabetes mellitus patients with nonalcoholic fatty liver disease and positive correlation with serum uric acid. *Cell Biochem Biophys* 2015; **72**: 643-7.

For peer review only

34. Liu Z, Que S, Zhou L, Zheng S. Dose-response relationship of serum uric acid with metabolic syndrome and non-alcoholic fatty liver disease incidence: a meta-analysis of prospective studies. *Sci Rep* 2015; **5**: 14325.

35 Cai W, Wu X, Zhang B, et al. Serum uric acid levels and non-alcoholic fatty liver disease in Uyghur and Han ethnic groups in northwestern China. *Arq Bras Endocrinol Metabol* 2013;**57**:617-22.

36. Zhou Y, Wei F, Fan Y. High serum uric acid and risk of nonalcoholic fatty liver disease: A systematic review and meta-analysis. *Clin Biochem* 2016; **49**: 636 – 42.

37 Parsons WH, Kolar MJ, Kamat SS, et al. AIG1 and ADTRP are atypical integral membrane hydrolases that degrade bioactive FAHFAs. *Nat Chem Biol* 2016;**12**:367-72.

38. Kolar MJ, Kamat SS, Parsons WH, et al. Branched Fatty Acid Esters of Hydroxy Fatty Acids Are Preferred Substrates of the MODY8 Protein Carboxyl Ester Lipase. *Biochemistry* 2016;**55**:4636-41.

39. Moraes-Vieira PM, Saghatelian A, Kahn BB. GLUT4 Expression in Adipocytes Regulates De Novo Lipogenesis and Levels of a Novel Class of Lipids With Antidiabetic and Anti-inflammatory Effects. *DIABETES* 2016;**65**:1808-15.

40. Zhu Q-F, Yan J-W, Gao Y, et al. Highly sensitive determination of fatty acid esters of hydroxyl fatty acids by liquid chromatography-mass spectrometry. *J Chromatogr B* 2017;**1061-1062**:34-40.

41. Lee J, Moraes-Vieira PM, Castoldi A, et al. Branched Fatty Acid Esters of Hydroxy Fatty Acids (FAHFAs) Protect against Colitis by Regulating Gut Innate and Adaptive Immune Responses. *J Biol Chem* 2016;**291**:22207-17.

42. Sitthi-amorn C, Poshyachinda V. Bias. *Lancet*. 1993;**342**: 286-8.

For peer review only

Table 1 Participant characteristics.

a. Subset

Indicators	Total	NAFLD	No NAFLD	*P* value
**n**	447	102 (22.8%)	345 (77.2%)	
**Age (years)**	44.3 ±11.9	44.6 ±10.8	44.0 ±12.1	0.609
**Age stage**	4.0±1.2	4.0±1.2	3.9±1.2	0.608
**Obesity**	235 (51.8%)	94	141	<0.001
**Body fat**	309 (68.1%)	89	220	<0.001
**Blood lipids**	87 (19.2%)	21	66	0.745
**Hypertension**	55 (12.1%)	23	32	0.003
**Male**	171 (38.3%)	64	107	<0.001
**Elevated liver enzymes**	18 (4.0%)	7	11	0.174

Age stages of 20-30, 30-40, 40-50, 50-60, ≥60 are 2, 3, 4, 5, 6.

b Total study population

Indicators	Total	NAFLD	No NAFLD	*P* value
**n**	1479	365 (24.7%)	1114 (75.3%)	
**Obesity**	809 (54.7%)	334	475	<0.001
**Body fat**	1003 (67.8%)	309	694	<0.001

For peer review only

**Blood lipid**	458 (30.9%)	163	295	<0.001
**High blood glucose**	71 (4.8%)	43	28	<0.001
**Hypertension**	314 (21.2%)	129	185	<0.001
**Abnormal uric acid**	83 (5.6%)	38	45	<0.001
**Male**	590 (39.8%)	214	376	<0.001
**Impaired liver function**	32 (0.2%)	12	20	0.142
**Elevated liver enzymes**	110 (0.7%)	53	57	<0.001

Conditions were defined as follows: obesity (BMI ≥ 24 kg/m^2), hypertension (blood pressure ≥ 140/90 mmHg or antihypertensive drug treatment), Body fat ratio ≥ 25 for women or ≥ 20 for men, blood triglycerides ≥ 1.7 mmol/L, blood fasting glucose ≥ 5.6 mmol/L, impaired liver function (positive HBsAg), liver enzyme elevation (AST ≥ 40 U/L or ALT ≥ 40 U/L), uric acid ≥ 357 μmol/L for women or ≥ 416 μmol/L for men.

Mean values are provided with standard deviation, unless otherwise noted as n (%). Differences between participants with and without NAFLD were evaluated with t-tests or the Wilcoxon-Mann-Whitney test for continuous variables and the chi-squared test for categorical variables. NAFLD, non-alcoholic fatty liver disease.

For peer review only

Table 2. NAFLD prevalence across age range.

**Age range (years)**	**Participants, n**	**Participants with NAFLD, n**	**NAFLD prevalence,%**	**U-test**
20-30	64	10	15.6	1.22
30-40	102	27	26.4	0.75
40-50	120	26	21.7	0.18
50-60	111	30	27.0	1.00
≥60	50	9	18.0	0.64
Total	447	102	22.8	1.64 (α=0.1)

NAFLD, non-alcoholic fatty liver disease.

For peer review only

Table 3. Multivariate adjusted models for liver disease subtypes.

a Subset

Variables	OR	*p* value
Sex (male)	3.484	<0.001
Obesity	11.738	<0.001
Body fat	2.285	0.033
Elevated liver enzyme	2.237	0.105
Hypertension	1.865	0.089

b The whole

Variables	OR	*p* value
Sex (male)	2.646	<0.001
Obesity	8.494	<0.001
Body fat	1.833	0.001
Blood lipid	1.340	0.046
Blood glucose	3.324	<0.001
Impaired liver function	1.859	0.094
Elevated liver enzyme	3.150	<0.001
Hypertension	1.451	0.017

Conditions were defined as follows: female sex=1, obesity (BMI ≥ 28 kg/m^2), hypertension (blood pressure ≥ 140/90 mmHg or antihypertensive drug treatment), body fat ratio ≥ 25 for women or ≥ 20 for men, blood fasting

For peer review only

glucose ≥ 6.1 mmol/L, liver enzyme elevation (AST ≥ 40 U/L or ALT ≥ 40 U/L)

NAFLD, non-alcoholic fatty liver disease.

Odds ratios and the corresponding p values derived from binary or multiple logistic regression analyses using SPSS software version 17.0 (SPSS Inc., Chicago, IL, USA)

For peer review only

Table 4. Metabolites associated with non-alcoholic fatty liver disease.

Metabolite	OR, *P* value	AUC, *P* value
**Uric acid**	2.755 <0.001	0.579 <0.001
**Sphingosine**	1.448 0.156	0.489 0.760
**OAHOA**	1.340 0.046	0.612 0.001

Conditions were defined: Sphingosine < 2 nmol/L, OAHOA (oleic acid -hydroxy oleic acid) < 5 nmol/L.

NAFLD, non-alcoholic fatty liver disease.

Odds ratios (OR), area under curve (AUC) and the corresponding p values derived from binary or multiple logistic regression, or ROC analyses using SPSS software version 17.0 (SPSS Inc., Chicago, IL, USA)

For peer review only

STROBE Statement—checklist of items that should be included in reports of observational studies

	Item No.	Recommendation	Page No.
**Title and abstract**	1	(*a*) Indicate the study's design with a commonly used term in the title or the abstract	1
		(*b*) Provide in the abstract an informative and balanced summary of what was done and what was found	2
**Introduction**			
Background/rationale	2	Explain the scientific background and rationale for the investigation being reported	4
Objectives	3	State specific objectives, including any prespecified hypotheses	5
**Methods**			
Study design	4	Present key elements of study design early in the paper	5
Setting	5	Describe the setting, locations, and relevant dates, including periods of recruitment, exposure, follow-up, and data collection	5,6,7,8
Participants	6	(*a*) *Cohort study*—Give the eligibility criteria, and the sources and methods of selection of participants. Describe methods of follow-up<br>*Case-control study*—Give the eligibility criteria, and the sources and methods of case ascertainment and control selection. Give the rationale for the choice of cases and controls<br>*Cross-sectional study*—Give the eligibility criteria, and the sources and methods of selection of participants	5,6
		(*b*)*Cohort study*—For matched studies, give matching criteria and number of exposed and unexposed<br>*Case-control study*—For matched studies, give matching criteria and the number of controls per case	
Variables	7	Clearly define all outcomes, exposures, predictors, potential confounders, and effect modifiers. Give diagnostic criteria, if applicable	6,7,8
Data sources/ measurement	8*	For each variable of interest, give sources of data and details of methods of assessment (measurement). Describe comparability of assessment methods if there is more than one group	6,7,8
Bias	9	Describe any efforts to address potential sources of bias	6
Study size	10	Explain how the study size was arrived at	5

Continued on next page

For peer review only

Quantitative variables	11	Explain how quantitative variables were handled in the analyses. If applicable, describe which groupings were chosen and why	7,8
Statistical methods	12	(*a*) Describe all statistical methods, including those used to control for confounding	8
		(*b*) Describe any methods used to examine subgroups and interactions	8
		(*c*) Explain how missing data were addressed	
		(*d*) *Cohort study*—If applicable, explain how loss to follow-up was addressed<br>*Case-control study*—If applicable, explain how matching of cases and controls was addressed<br>*Cross-sectional study*—If applicable, describe analytical methods taking account of sampling strategy	8
		(*e*) Describe any sensitivity analyses	
**Results**			
Participants	13*	(a) Report numbers of individuals at each stage of study—eg numbers potentially eligible, examined for eligibility, confirmed eligible, included in the study, completing follow-up, and analysed	8,9
		(b) Give reasons for non-participation at each stage	
		(c) Consider use of a flow diagram	
Descriptive data	14*	(a) Give characteristics of study participants (eg demographic, clinical, social) and information on exposures and potential confounders	8,9
		(b) Indicate number of participants with missing data for each variable of interest	
		(c) *Cohort study*—Summarise follow-up time (eg, average and total amount)	
Outcome data	15*	*Cohort study*—Report numbers of outcome events or summary measures over time	
		Case-control study—Report numbers in each exposure category, or summary measures of exposure	
		Cross-sectional study—Report numbers of outcome events or summary measures	8,9
Main results	16	(*a*) Give unadjusted estimates and, if applicable, confounder-adjusted estimates and their precision (eg, 95% confidence interval). Make clear which confounders were adjusted for and why they were included	9,10,11
		(*b*) Report category boundaries when continuous variables were categorized	
		(*c*) If relevant, consider translating estimates of relative risk into absolute risk for a meaningful time period	

Continued on next page

For peer review only - http://bmjopen.bmj.com/site/about/guidelines.xhtml

Other analyses	17	Report other analyses done—eg analyses of subgroups and interactions, and sensitivity analyses	
**Discussion**			
Key results	18	Summarise key results with reference to study objectives	11,12,13
Limitations	19	Discuss limitations of the study, taking into account sources of potential bias or imprecision. Discuss both direction and magnitude of any potential bias	14
Interpretation	20	Give a cautious overall interpretation of results considering objectives, limitations, multiplicity of analyses, results from similar studies, and other relevant evidence	11,12,13
Generalisability	21	Discuss the generalisability (external validity) of the study results	14
**Other information**			
Funding	22	Give the source of funding and the role of the funders for the present study and, if applicable, for the original study on which the present article is based	15

*Give information separately for cases and controls in case-control studies and, if applicable, for exposed and unexposed groups in cohort and cross-sectional studies.

Note: An Explanation and Elaboration article discusses each checklist item and gives methodological background and published examples of transparent reporting. The STROBE checklist is best used in conjunction with this article (freely available on the Web sites of PLoS Medicine at http://www.plosmedicine.org/, Annals of Internal Medicine at http://www.annals.org/, and Epidemiology at http://www.epidem.com/). Information on the STROBE Initiative is available at www.strobe-statement.org.

For peer review only

For peer review only - http://bmjopen.bmj.com/site/about/guidelines.xhtml
